# Wintertime Formation of Large Sulfate Particles in China and Implications for Human Health

**DOI:** 10.1021/acs.est.3c05645

**Published:** 2023-11-01

**Authors:** Qianru Zhang, Yuhang Wang, Maodian Liu, Mingming Zheng, Lianxin Yuan, Junfeng Liu, Shu Tao, Xuejun Wang

**Affiliations:** † Ministry of Education Laboratory of Earth Surface Processes, College of Urban and Environmental Sciences, Peking University, Beijing 100871, China; ‡ School of Earth and Atmospheric Sciences, Georgia Institute of Technology, Atlanta, Georgia 30332, United States; § School of the Environment, Yale University, New Haven, Connecticut 06511, United States; ∥ School of Chemical and Environmental Engineering, Wuhan Polytechnic University, Wuhan 430023, China; ⊥ Hubei Environmental Monitoring Center, Wuhan 430072, China

**Keywords:** winter haze, sulfate aerosol, micron-sized distribution, human health, climate

## Abstract

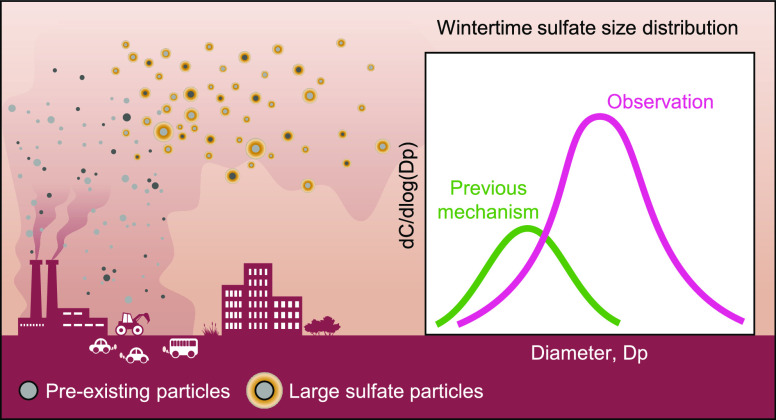

Outdoor air pollution causes millions of premature deaths annually worldwide. Sulfate is a major component of particulate pollution. Winter sulfate observations in China show both high concentrations and an accumulation mode with a modal size >1 μm. However, we find that this observed size distribution cannot be simulated using classical gaseous and aqueous phase formation (CSF) or proposed aerosol-processing formation (APF) mechanisms. Specifically, the CSF simulation underestimates sulfate concentrations by 76% over megacities in China and predicts particle size distributions with a modal size of ∼0.35 μm, significantly smaller than observations. Although incorporating the APF mechanism in the atmospheric chemical model notably improves sulfate concentration simulation with reasonable parameters, the simulated sulfate particle size distribution remains similar to that using the CSF mechanism. We further conduct theoretical analyses and show that particles with diameters <0.3 μm grow rapidly (2–3 s) to 1 μm through the condensation of sulfuric acid in fresh high-temperature exhaust plumes, referred to as in-source formation (ISF). An ISF sulfate source equivalent to 15% of sulfur emissions from fossil fuel combustion largely explains both observed size distributions and mass concentrations of sulfate particles. The findings imply that ISF is a major source of wintertime micron-sized sulfate in China and underscore the importance of considering the size distribution of aerosols for accurately assessing the impacts of inorganic aerosols on radiative forcing and human health.

## Abstract 2

The in-source formation mechanism explains not only high sulfate mass concentrations but more importantly the formation of large micron-sized sulfate particles observed in winter in China.

## Introduction

Air pollution is a global concern.^1−3^ Sulfate is a major component of atmospheric PM_2.5_ (fine particles with an aerodynamic diameter <2.5 μm), which has adverse effects on visibility, air quality, and public health,^4,5^ especially during winter haze events.^6,7^ Sulfate aerosols also have significant impacts on Earth’s radiation balance and climate by scattering solar radiation and serving as cloud condensation nuclei (CCN).^8^ Despite its importance, atmospheric models generally underestimate sulfate concentrations under winter haze conditions in China,^9−11^ and the formation mechanism of sulfate is debatable.^12−15^ Most models include classical gaseous and aqueous phase sulfate formation (CSF) pathways, such as the oxidation of sulfur dioxide (SO_2_) by hydroxyl radicals in the gas phase and by dissolved oxidants (hydrogen peroxide, ozone, nitrogen dioxide, and oxygen catalyzed by transition-metal ions) in cloud water.^4,16^ However, these CSF mechanisms cannot explain the abundant sulfate observed in winter in China.^9−11,17^ A few studies suggested that reactions occurring in fog and clouds might have major contributions to the formation of sulfate.^18,19^ However, fog and clouds infrequently occur during winter haze events,^20,21^ and researchers have found that in-cloud reactions are not a key pathway for sulfate formation during winter haze episodes.^9,11^

Some studies proposed that aerosol-processing formation (APF), that is, oxidation of SO_2_ by various oxidants on aerosols, has the potential to explain the missing sulfate over China in atmospheric models.^9,11^ Recent studies further proposed and highlighted several APF routes with specific oxidants and attempted to parametrize the reaction rates associated with APF.^13,22−24^ However, these studies did not take into account the influence of APF on the sulfate size distribution. In comparison to the accumulation mode at a modal size of 0.4–0.8 μm observed in North America,^25−28^ the dry sulfate in wintertime China appears significantly larger, with a modal size of 0.6–1.4 μm.^29−32^ New research noticed the potential contribution of plumes in the formation of atmospheric particulate matter,^33−35^ whereas the importance of this mechanism to the formation of large sulfate particles under polluted background remains to be deciphered. Furthermore, observations near coal-fired power plant exhaust in China showed that a large fraction of sulfur emissions are in the form of SO_3_,^36−39^ which is produced through the oxidation of SO_2_ during combustion and can also be formed in the exhaust plumes.^12^ Unlike SO_2_, SO_3_ is converted to sulfuric acid virtually instantly in the troposphere.^40^ Thus, we hypothesize that the condensation of sulfuric acid in fresh high-temperature exhaust plumes onto pre-existing atmospheric particles can quickly produce micron-sized particles. We refer to the process of sulfate particle production from fresh plumes hereafter as in-source formation (ISF), which may be the missing mechanism of sulfate formation in China.

In this study, we aim to answer the question of whether ISF can explain both the observed high mass concentrations and large-size distributions of sulfate in the winter in China. Our investigation involves simulations of sulfate mass concentrations and size distributions using CSF, APF, and ISF, with an emphasis on the formation of large wintertime sulfate particles across China using the Weather Research and Forecasting model coupled with Chemistry (WRF-Chem) model with detailed aerosol microphysics. To avoid any confusion, we specifically use the term “APF” instead of the commonly used term “heterogeneous sulfate formation” as ISF can also be heterogeneous. We leverage a wide range of diverse observations to thoroughly assess the robustness of our simulations and constrain our results. We pay particular attention to aerosol size measurements, which were not generally considered in previous studies of the wintertime sulfate formation processes. Through the development of a theoretical model and conducting sensitivity experiments, we examine the feasibility of large sulfate particle formation via ISF. Our study reveals substantial disparities in particulate size distributions predicted by APF and ISF, potentially challenging the current understanding of the effects of aerosols on radiative forcing and public health implications.

## Methods

### Observations

Hourly observations of surface PM_2.5_ chemical compositions and gaseous pollutants were monitored at an urban site in Wuhan, central China (30.53°E, 114.36°N, denoted by the blue dot in Figure 1d) from December 2014 to February 2015. Water-soluble ions (including sulfate, nitrate, and ammonium) in PM_2.5_ and SO_2_ were measured using an online ion chromatography analyzer (MARGA-ADI 2080) and ultraviolet fluorescence equipment, respectively.^41^ For MARGA measurements, the sampling flow rate of air was 16.7 L min^–1^. The recovery rates were controlled to be >95%, and the relative standard deviation of the sampling flow was calibrated to <5%. Detailed quality assurance and quality control at the site were performed as described in our previous study.^41^ Wuhan and its surrounding areas have suffered from severe air pollution in recent years, which has attracted increasing attention.^42^ The site in Wuhan is a typical urban site in central China. Additionally, the time resolution of these data was particularly high, and the data fully covered the entire winter season. Currently, hourly measurements of PM_2.5_ components (such as SO_4_^2–^, NO_3_^–^, NH_4_^+^) are not available at other locations in China. Thus, we employed these data to evaluate the model performance.

**Figure 1:**
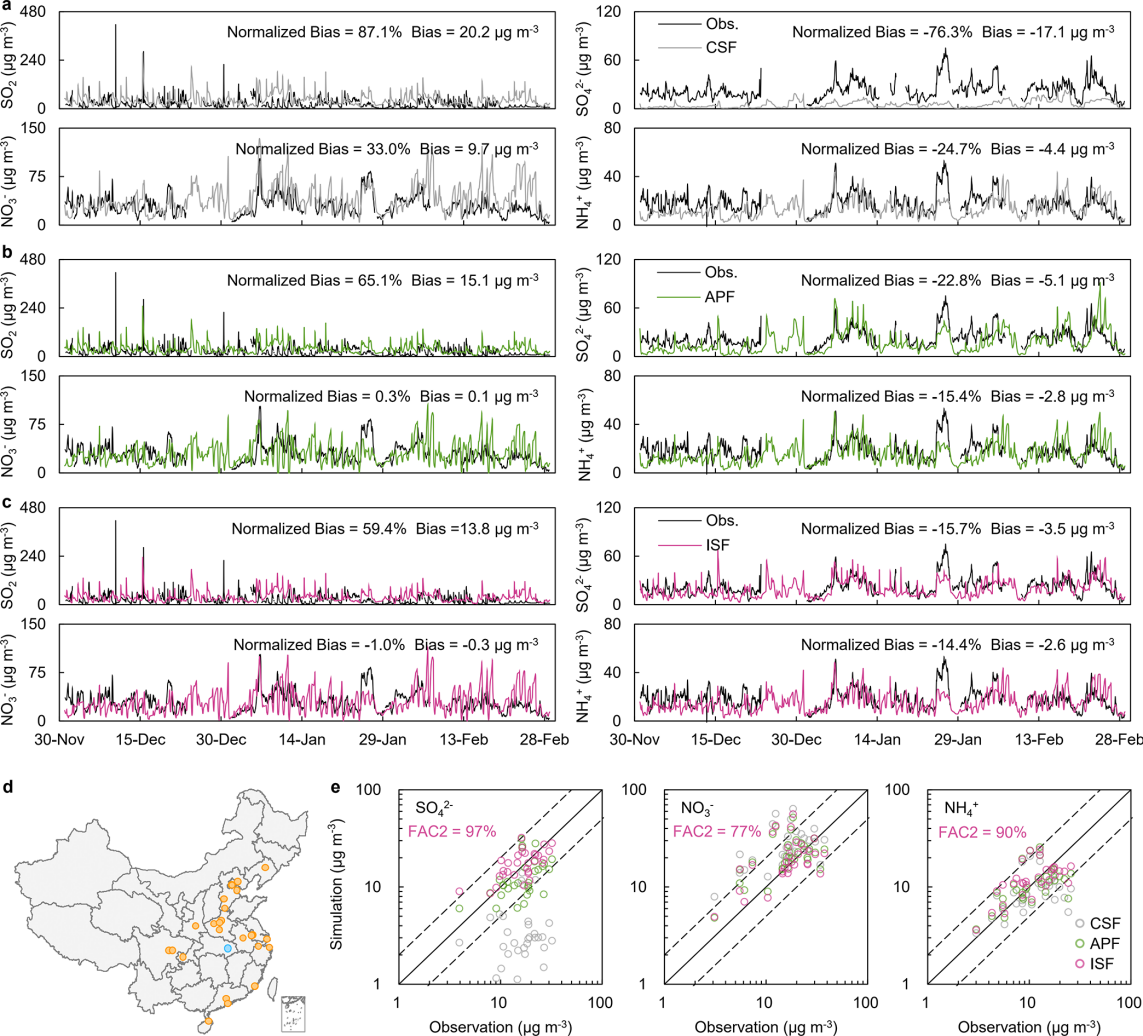
Observed and simulated wintertime air pollutants over China. (a–c) Observed and simulated hourly SO_2_, sulfate, nitrate, and ammonium concentrations at the Wuhan site from December 2014 to February 2015. The bias and normalized bias were calculated for each set of observed and simulated data. “Obs.” denotes the observation. CSF, APF, ISF are different mechanisms of sulfate formation. (CSF: classical gaseous and aqueous phase sulfate formation; APF: aerosol-processing formation; ISF: In-source formation.) See Methods for detailed settings of simulations. (d) Location of the Wuhan site (denoted by the blue dot) and other 25 observation stations (denoted by orange dots) over China. (e) Comparison between observed and simulated sulfate, nitrate, and ammonium concentrations for 31 sets of data from 25 observation stations around China from December 2014 to February 2015. Observed data were monthly average data or several days’ average data (listed in Supporting Information Table S3). Here, the simulated data were averaged over the period corresponding to each observation time. Two dashed lines are 1:2 and 2:1 lines. The FAC2 index here was calculated for the ISF simulation, which represents the fraction of simulations within a factor of 2 of observations.

**Figure 2:**
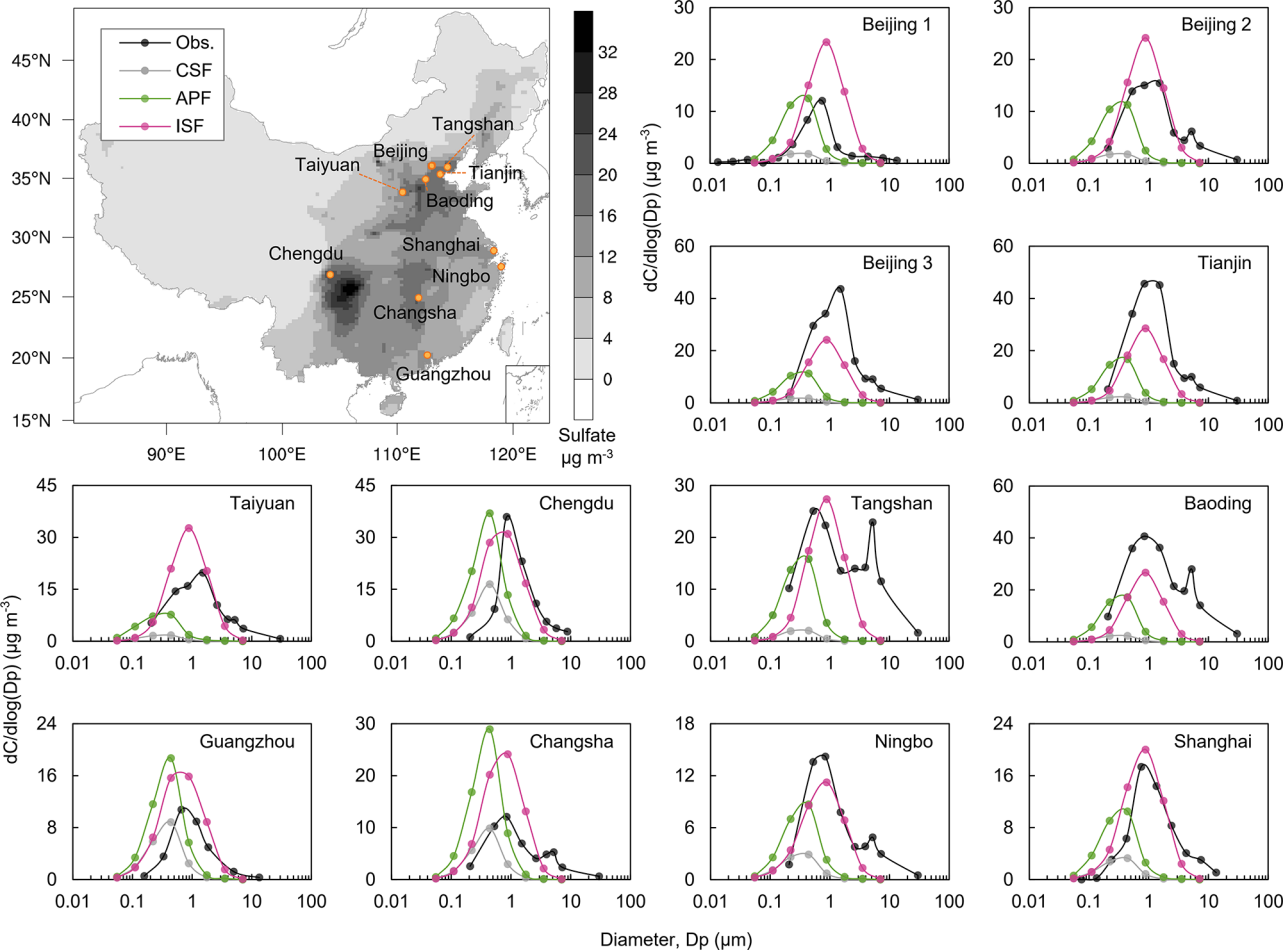
Observed and simulated wintertime sulfate size distribution over China. The panel in the upper left corner shows the spatial distribution of average sulfate concentrations over China from December 2014 to February 2015 in the ISF simulation. The orange dots on the map denote the 12 observation sites. If the sampling time of one site is within the simulation period, the mean value of the simulated data corresponding to observation time was used to compare with the observation data (obs.) (listed in Supporting Information Table S4); otherwise, the three-month average values were employed to compare with the observation data. (CSF: classic gaseous and aqueous phase sulfate formation; APF: aerosol-processing formation; ISF: In-source formation).

We also collected several other types of wintertime observations across China to compare with the simulation results: (1) mass concentration data of 31 sets of sulfate, nitrate, and ammonium at 25 stations over China (denoted by orange dots in Figure 1d) within the simulation period were obtained from the literature (Supporting Information Table S3); (2) long-term (regular multiple samplings over at least 7 days) mass size distribution data of sulfate, nitrate, and ammonium were compiled from published articles (denoted by orange circles in Figure 2; Supporting Information Table S4); (3) hourly observations of SO_2_ and PM_2.5_ and monthly meteorological data over China were obtained from the China Ministry of Ecology and Environment (http://106.37.208.233:20035/) and the China Meteorological Data Service Centre (http://data.cma.cn), respectively. The inorganic aerosol size-distribution data were selected based on the following criteria: the sampling site was a nonbackground air site, sampling time was relatively new (between 2010 and 2017), the number of samples was relatively large, and the sample period was not affected by special events (such as dust storms or emission reduction for social events). The mass size distribution data of inorganic aerosols were mostly measured by using the following method. First, size-resolved aerosols were sampled using a multistage cascade impactor (such as the MOUDI sampler or Andersen sampler shown in Supporting Information Table S4). Previous studies consistently showed that despite potential variations in ambient humidity and aerosol liquid water content between daytime and nighttime, for example, the aerosol liquid water content in PM_2.5_ during winter nights in Beijing were more than 2 times higher than daytime levels,^43^ the size distributions of sulfate sampled at the same location from day and night were very similar and the modal size of the size distribution remained relatively consistent.^44,45^ Thus, following previous studies, we think the aerosols are basically under dry conditions during sampling.^44,46^ Then, composition analysis of water-soluble ions in each stage was carried out, and the mass size distribution data of water-soluble ions (including sulfate, nitrate, and ammonium) were finally obtained.

### Atmospheric Model and Simulations

WRF-Chem is a fully online coupled meteorology–chemistry model. It has long been used to simulate regional air quality and evaluate meteorology–chemistry interactions.^47,48^ Here, we used WRF-Chem version 3.9 to simulate the emission, transport, chemical reactions, and dry and wet depositions of chemical species in this study.^49^ There are several options for parametrizing the physical and chemical processes in the model. In the WRF-Chem default model, the primary sulfate emissions can be incorporated in the model.^50,51^ The amount of primary sulfate emissions depends on the emission inventory used. For the chemical mechanisms, we used the Carbon Bond Mechanism version Z (CBMZ) for the gas-phase chemical mechanism^52^ and the Model for Simulating Aerosol Interactions and Chemistry (MOSAIC) with eight bins (Supporting Information Table S1) for the aerosol simulations.^53^ MOSAIC includes all major aerosol species and simulates aerosol chemical and physical processes such as nucleation, condensation, coagulation, thermodynamics, and phase equilibrium. More details about the MOSAIC aerosol model are presented in Supporting Information Text S1. Chemical initial and boundary conditions of WRF-Chem were obtained from simulations of the Community Atmosphere Model with Chemistry (CAM-Chem) model.^54^ For every simulation and sensitivity experiment, the WRF-Chem model was run for 3 months from December 2014 to February 2015, covering the entire winter season in China. Observations for the same period were used to evaluate the model simulations. The model results for the first 5 days were excluded as the model spin-up for each monthly simulation.^55^ The simulation domain covered mainland China (Supporting Information Figure S1), with a horizontal resolution of 30 km and 30 vertical levels. Detailed configurations following published literature^56,57^ are listed in Supporting Information Table S2.

For anthropogenic emissions, we employed the Multiresolution Emission Inventory for China (MEIC) 2014–2015 inventory, which provides the monthly emissions of major air pollutants from five major emission sectors. The MEIC inventory uses more data specific to China compared to global inventories and has been widely used in air quality research.^58^ In this emission inventory, no primary sulfate emissions were included. To enhance the accuracy of emissions data in China, we made some adjustments. Specifically, we replaced the ammonia (NH_3_) emission inventory with the PKU-NH_3_ 2015 inventory, which was developed based on a process-based method to better represent the temporal and spatial distributions of NH_3_ emissions in China.^56^ Additionally, we replaced residential SO_2_ emissions with the PKU-FUEL 2014 inventory, compiled based on the latest nationwide survey of residential fuel consumption in China.^59^ The mosaic Asian anthropogenic emission inventory (MIX inventory) was used for the anthropogenic emissions outside China.^60^ We considered hourly and vertical variations in anthropogenic emissions based on emission sectors. Biogenic emissions were calculated online using the Model of Emissions of Gases and Aerosols from Nature (MEGAN) module,^61^ and hourly fire emissions were provided by the Fire Inventory from the NCAR (FINN) model.^62^

Two additional sulfate sources (APF and ISF) were considered in this study. For the APF mechanism, following the existing code of the heterogeneous uptake of dinitrogen pentoxide (N_2_O_5_) in the model,^63^ we parametrized the process of the heterogeneous uptake of SO_2_ on aerosol particles and subsequent oxidation by oxidants to form sulfates in the MOSAIC module of the WRF-Chem model. The reaction was assumed to be irreversible, and the uptake of SO_2_ by aerosol particles was a first-order process, as in a previous work^63^
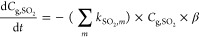
1where *C*_g,SO_2__ are the concentrations of gaseous SO_2_ in the ambient air (mol m^–3^). β is a hypothetical ratio that ensures that the total amount of sulfate produced by APF and ISF is the same in China for each month. Here, we introduce a parameter, β, such that the size distribution comparison using different mechanisms is analyzed under the condition of comparable sulfate mass concentrations. In this manner, the effect of sulfate mass concentration difference on size distribution difference was minimized. In this study, the three-month average value of β was 1.10, and the deviation of the monthly value of β from the average was within 0.09. The calculation process of β in the APF simulation is presented in Supporting Information Text S2. *k*_SO_2_,*m*_ is the first-order mass transfer coefficient for SO_2_ in bin *m* (s^–1^) and is parametrized as
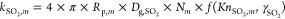
2where *R*_p,*m*_ is the particle radius (cm), *D*_g,SO_2__ is the gas diffusivity of SO_2_ (cm^2^ s^–1^), *N*_*m*_ is the particle number density in bin *m* (cm^–3^), and *f*(*Kn*_SO_2_,*m*_, γ_SO_2__) is the transition regime correction factor (discussed in eq 4). In the parametrization, the limiting step was set as the uptake of SO_2_, which is controlled by the reactive uptake coefficient γ_SO_2__.^11,63^ A wide range of γ_SO_2__ values (0.6 × 10^–5^ to 0.1) has been previously reported.^9,11^ Here, we employed a constant γ_SO_2__ of 1 × 10^–5^, which shows a reasonable agreement with the observations.

We introduced the ISF mechanism into the model by considering multiple types of observational data, emission inventory uncertainties, and a series of model sensitivity analyses. Bottom-up estimates generally suggested that ∼5% of sulfur emissions from China’s coal-fired power plants are in the form of SO_3_,^64^ while observations from the coal-fired power plant exhaust in stacks suggested that SO_3_ emissions account for 20% of total sulfur emissions.^36−39^ Most bottom-up estimates were based on in-stack measurements, but the actual amount of SO_3_ in fresh exhaust plumes may be higher since SO_2_ can be oxidized to SO_3_ in the exhaust plumes.^12^ According to sensitivity experiments, the observed sulfate concentrations in China were well reproduced when 10%–15% of sulfur was emitted in the form of SO_3_. For sulfate size distribution, field measurements in China showed a sulfate modal size of 0.7–1.1 μm during the combustion of coal and wood,^65^ while our sensitivity experiments showed that a sulfate modal size of 0.8–1 μm emitted in the model can explain well the observed sulfate size distributions (Figure 2). We assumed that the ISF source of sulfate was 15% of the sulfur emissions from all fossil fuel combustion sectors with a modal size of 0.9 μm and a geometric standard deviation of 2 μm according to the published literature.^4^ It is worth noting that the ISF mechanism proposed in this study is distinct from primary sulfate emissions in terms of their physical origin. Primary sulfate is conventionally attributed to combustion processes, whereas ISF sulfate pertains to sulfate formation within the plume.

### Theoretical Analysis of the ISF Particle Size Growth

To examine the particle growth process during ISF inside or near smokestacks, we employed MATLAB R2020b to develop a theoretical model to calculate the particle size growth over time. There are many pre-existing particles in the polluted atmosphere that provide abundant surface areas for the condensation of sulfuric acid vapor in the exhaust. Following a previous study,^4^ the rate of sulfuric acid vapor condensing onto the surface of particle *I* (kg s^–1^) was calculated using the following equation
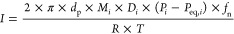
3where *d*_p_ is the diameter of the particle (m), *M*_*i*_ is the molar mass of sulfuric acid vapor (98 × 10^–3^ kg mol^–1^), *D*_*i*_ is the diffusion coefficient of sulfuric acid vapor in the air (m^2^ s^–1^), and *P*_*i*_ is the vapor pressure of sulfuric acid in the gas phase (Pa), calculated from the concentration of SO_3_ in the flue gas and the temperature of the flue gas. Based on the observations of SO_3_ and temperature,^12,38^ these two variables were set to 5 mg m^–3^ and 50 °C, respectively. *P*_eq,*i*_ is the equilibrium vapor pressure on the particle surface. As sulfuric acid is nonvolatile, *P*_eq,*i*_ was set to zero during the calculation. *R* is the gas constant (8.314 J K^–1^ mol^–1^), and *T* is the temperature of the surface of the particle (*K*), which was set equal to the temperature of ambient air (0 °C). *f*_n_ is the transition regime correction factor expressed as^53,66^
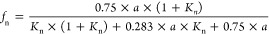
4where *K*_n_ is the Knudsen number, defined as *K*_n_ = (2 × λ)/*d*_p_ (where λ is the mean free path),^53^ and *a* is the mass accommodation coefficient with a value of 0.65.^67^ Finally, according to the law of conservation of mass, the growth rate of a particle can be expressed as follows
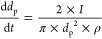
5where ρ is the density of the particle (kg m^–3^).

### Radiative Forcing and Health Impact

We calculated the radiative forcing of aerosols using the WRF-Chem model Rapid Radiative Transfer Model for General Circulation Models (RRTMG) longwave and shortwave radiation scheme.^68^ For the direct radiative effects, we calculated the all-sky direct radiative effects for different size distributions. At every time step, we kept all of the meteorological parameters, cloud properties, surface properties, and aerosol mass loadings the same and used the APF or ISF size distributions of sulfate, nitrate, and ammonium in the model. We then calculated the aerosol radiative properties (aerosol optical depth, single-scattering albedo, and asymmetry factor) with two different size distributions and obtained two sets of radiation flux data at the surface. In addition to direct radiative forcing, the size distribution of aerosols can influence the formation of CCN and aerosol-cloud indirect effects.^69^ However, in the WRF-Chem model, aerosols, clouds, and meteorologies are coupled and affect each other, making the indirect effects challenging to quantify. Here, we calculated the CCN concentration and total radiative forcing (containing all of the effects of aerosols) in the APF and ISF simulations with the same total sulfate mass emission in China but different particle size distributions of secondary inorganic aerosols. Finally, we compared the results of the two simulations and computed the impact of different aerosol size distributions on the total radiative forcing at the surface.

The International Commission on Radiological Protection (ICRP) particle deposition model was employed to evaluate the size-resolved health impacts on humans,^70^ which provides size-resolved particle deposition fractions in different regions (head airway and nose region, tracheobronchial region, and alveolar region) in the human respiratory system. We applied fitted equations from the ICRP model (Supporting Information Figure S2), which was calculated by averaging the data from different exercise levels for men and women.^71^ Because the original model did not account for the moisture absorption and growth of particles in the lungs, we used the approach of Kodros et al. to calculate the hygroscopic growth of particles in the lungs using the hygroscopicity parameter computed with eq 6–10^72^
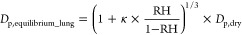
6where *D*_p,equilibrium_lung_ is the particle size after water uptake and equilibrium in the lungs, *D*_p,dry_ is the diameter of the dry particle (m), RH is the relative humidity in the lungs, and κ is the hygroscopicity parameter.^73^ RH and the temperature in the lungs were set to be 99.5% and 37 °C, respectively, following the published literature.^74^ The total particle κ value was calculated as the volume-weighted average of all individual species.^72^ The time required for the particles to reach equilibrium τ (s) in the lungs was calculated using eq 7(72)
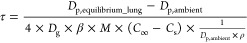
7where *D*_p,ambient_ is the initial particle diameter in the ambient environment (m), *D*_g_ is the diffusion coefficient of water vapor (m^2^ s^–1^), β is the correction factor, *M* is the molar mass of water vapor (18 g mol^–1^), ρ is the density of particle (g m^–3^), and *C*_∞_ and *C*_s_ are the water vapor concentration in the lung and at the particle surface (mol m^–3^), respectively. Equations 8 and 9 were used to compute the equilibrium particle size in the lungs^72^
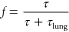
8
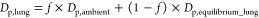
9where *D*_p,lung_is the diameter of the particle in the lung (m) and τ_lung_ is the residence time in the lung (2.5 s).^72^ Finally, the particle diameter associated with deposition in the human body *D*_p,deposition_, which is the aerodynamic diameter after considering the water uptake in the lung, was expressed as^72^
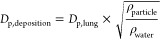
10where ρ_particle_ and ρ_water_ are the density of the particle (g cm^–3^) and water (1 g cm^–3^), respectively.

## Results and Discussion

### High Concentrations and Large Sizes of Sulfate Particles in Wintertime in China

We found that neither CSF nor APF could explain the observed mass concentrations and size distributions of sulfate in China. The WRF-Chem simulation results show that the standard CSF mechanism substantially underestimates sulfate concentrations in winter. For instance, the simulated sulfate concentrations from December 2014 to February 2015 at the Wuhan station (mean: 5.4 μg m^–3^; range: 0.3–22.9 μg m^–3^) were much lower than the observations (hourly data, sample size *n* = 1764; 22.4 μg m^–3^; 3.0–75.0 μg m^–3^) (Figure 1a). From the hourly observations, it was found that CSF accounted for only 24% of the observed near-surface sulfate concentrations in Wuhan. Additionally, significant model biases were observed in other megacities in China, with a normalized bias of −76% (Figure 1e). These findings align with previous studies that utilized alternative models.^10,22,75,76^ Furthermore, we found that CSF greatly underestimated the sulfate particle size in China. The observed size distributions at the different sites in winter showed consistent characteristics (Figure 2, Supporting Information Figure S3), with an accumulation mode at a median modal size of 0.91 μm (0.64–1.39 μm, min to max). At a few sites, we also observed a small peak in the coarse mode, which is likely attributable to the sulfate formation on dust particles.^77^ In contrast, the simulation results using CSF revealed particle size distributions characterized by an accumulation mode with a median modal size of 0.35 μm (ranging from 0.34 to 0.46 μm), which was more than two times smaller than the observed values. Under CSF, in-cloud and gas-phase oxidation are the primary pathways of sulfate production,^4^ suggesting that the sulfate mechanism overlooked in the model may also contribute to the formation of large sulfate particles.

When compared to that of CSF, the utilization of APF demonstrates a substantial enhancement in predicting sulfate concentrations. This is evidenced by a notable reduction in the normalized biases of sulfate concentrations from −76% to −23% at the Wuhan site (Figure 1b). These improvements are further supported by results obtained from the other 25 stations across China, where APF exhibits a higher degree of accuracy in simulating sulfate concentrations (Figure 1e). Specifically, 84% of the simulation data fall within a factor of 2 of the observations when employing the APF mechanism, in contrast to only 23% when using CSF. At these sites, simulations of nitrate, ammonium, and SO_2_ using the APF mechanism were also ameliorated (Figure 1b,e). However, we found that the APF could not explain the observed sulfate size distributions. The APF mechanism did not obtain increased sulfate particle sizes with an accumulation mode at a median modal size of 0.37 μm (0.35–0.44 μm, Figure 2, Supporting Information Figure S3), and this result was similar to that of CSF.

The APF mechanism occurs at the surface of the suspended aerosol particles, while the sulfate production rate of the APF reaction is proportional to the aerosol surface area concentration.^78^ In this study, aerosol surface area distributions of aerosols in APF simulation showed a unimodal distribution and peaked at ∼0.2 μm (Supporting Information Figure S4), which was similar to the typical urban aerosol surface distributions presented in the previous study.^4^ Under this mechanism, high aerosol surface area concentrations were mainly located in the range of 0.1–0.4 μm diameter, and aerosol surface area concentration limited the APF reactions;^78^ thus, we posit that this mechanism might be unable to capture the observed sulfate size distribution.

γ is also a key parameter for the APF reaction, which may vary with ambient temperature, humidity, etc. Based on previous studies^9,11^ and sensitivity analysis, we adopt a constant γ, which may introduce uncertainties on the quantity of sulfate particle production but not on the sulfate size distribution. It is unlikely that γ in the APF reactions varies significantly with the aerosol particle size because many meteorological variables affecting γ (e.g., temperature and relative humidity) of different particle sizes in the same location are the same. At present, observations regarding this issue are scant, and future research is needed. Further, our result of the APF simulation was consistent with the analysis by Meng and Seinfeld,^79^ who suggested that large particles produced from fog evaporation may be a major source of aerosols with a size of 0.7 μm in California. While the modal sizes of sulfate particles are mostly >0.7 μm in the observations across China (Figure 2), fog infrequently occurs during winter haze events and is not a major factor in China.^20^ Thus, APF is presumably not the primary mechanism for the missing sulfate in China.

### Large Sulfate Particles Explained by the In-Source Formation

**Figure 3:**
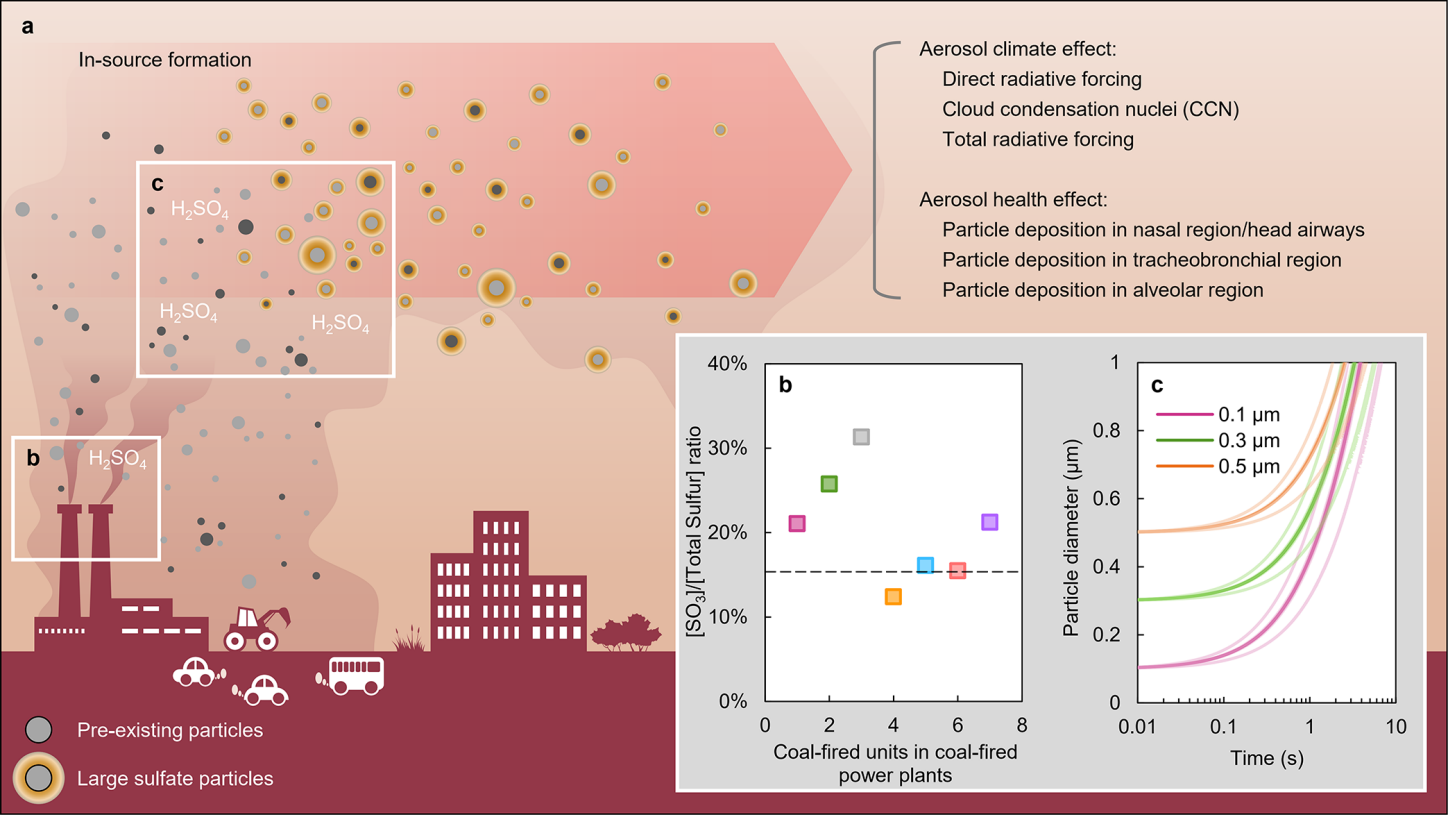
ISF under polluted conditions. (a) Schematic diagram showing the ISF of large sulfate particles. (b) Molar ratios of SO_3_ to total sulfur (SO_3_ + SO_2_) observed at the stack outlets of coal-fired power plants in China.^36−39^ The black dashed line represents the ratio used in this study (15%). (c) Increases in particle diameter with time due to the condensation of sulfuric acid. Three different initial particle sizes were considered at 0.1, 0.3, and 0.5 μm. Three thin colored curves are the sensitivity results with ambient temperature (±5 °C), flue gas temperature (±5 °C), and SO_3_ concentrations (±2 mg m^–3^).

We found that ISF largely explained both the observed mass concentrations and size distributions of sulfate in China. There are many potential pathways for the ISF. Here, we provide detailed analysis of the H_2_SO_4_ condensation pathway. However, for the other pathways, we presented only some speculations and hypotheses. Further research is needed in the future to investigate these potential pathways in more detail. The general process of H_2_SO_4_ condensation for ISF is shown in Figure 3a. First, a nontrivial fraction of fuel sulfur is oxidized to SO_3_ during combustion from various sectors and in the subsequent exhaust plumes. We conducted a meta-analysis of previous observations of coal-fired power plant exhausts^36−39^ and confirmed that a molar fraction of 12%–31% of sulfur emissions are in the form of SO_3_, and the rest is SO_2_ (Figure 3b). Previous field observations reported that the SO_3_ could also be formed via the oxidation of SO_2_ in the exhaust plumes of coal-fired power plants.^12,33^ Combustion sources other than coal-fired power plants also emit significant amounts of SO_3_ that produce atmospheric sulfates such as industrial boilers and residential combustion.^12,80^ Second, the high concentration of water vapor in the exhaust converts SO_3_ to sulfuric acid. Unlike SO_2_, SO_3_ converts almost instantly to sulfuric acid by reacting with atmospheric water vapor (1 × 10^–4^ s at 273 K with 0.3% water vapor).^40^ Finally, as a high-temperature exhaust plume mixes with the ambient atmosphere, the cooling process rapidly decreases the saturation vapor pressure of sulfuric acid and water vapor, leading to condensation onto existing atmospheric aerosols, the number concentration of which is usually high in wintertime China.^81^

To analyze the impact of ISF on the formation of large sulfate particles, we utilized a theoretical model that focuses on the rapid growth of these particles through the condensation of sulfuric acid onto small pre-existing particles. According to previous observations, the initial diameters of the pre-existing particles in the accumulation mode were set from 0.1 to 0.5 μm,^4^ and the exhaust plume was assumed to have an SO_3_ concentration of 5 mg m^–3^.^38^ The theoretical model simulation result showed that the pre-existing particles quickly grow to a size of 0.9 μm in 2.0–3.3 s (Figure 3c), suggesting that the observed SO_3_ concentrations are sufficiently high that sustained exposure to this concentration of SO_3_ can grow small pre-existing atmospheric particles to the observed range of sulfate aerosols (Supporting Information Figure S3). The aerosol number concentration comparison is also important for determining the mechanism; however, there is a lack of relevant observations. For more details, please refer to the discussion in Supporting Information Text S3.

We conjecture that another potential pathway for ISF is the formation and evaporation of large water droplets, which can promote the generation of large particles.^79^ The rapid cooling of water vapor can result in the formation of water droplets, providing a surface for the adsorption of sulfuric acid. As more ambient air mixes into the exhaust, the relative humidity decreases, and the water droplets evaporate, leaving behind large sulfate particles.^82^ In winter, lower ambient air temperature can result in more serious white plumes and likely enhance the formation of large sulfate particles.^83^ We hypothesize that the third potential pathway involves the catalytic oxidation of SO_2_ to sulfuric acid by transition metals.^13^ The high temperature, acidity, and water content of the exhaust can greatly accelerate catalytic SO_2_ oxidation compared to that in the ambient atmosphere.^13,84^ It is possible that similar heterogeneous processes occurring on the hot surface of a smokestack coated with transition metals could explain the significant portion of SO_3_ observed in coal-fired power plant exhaust. In the future, additional high-precision and multivariate simultaneous measurements (including particle number and mass size distributions as well as size-resolved chemical composition) are needed in the plumes and surrounding areas in China to enhance our understanding of the processes in the ISF mechanism.

By introducing the ISF into the atmospheric model in light of the observations and conducting simulations, we found that, compared with CSF, ISF greatly ameliorated the simulation of sulfate concentrations (normalized biases, −76% and −16% for CSF and ISF at the Wuhan site, respectively; Figure 1c). Furthermore, observations of sulfate from other megacities across China supported the effectiveness of ISF, with 97% of the simulation data falling within a factor of 2 of the observations under the ISF mechanism and normalized biases between simulations and observations at the 25 sites is 4% (Figure 1e). While the simulated sulfate concentrations at the Wuhan site in the ISF simulation were slightly lower than the observations, ISF was able to capture the overall sulfate concentration variations and performed well at the other 25 sites across China. The discrepancy at the Wuhan site may be partly attributed to the coarse resolutions of the emission inventories and model simulations, which can introduce averaging effects and uncertainties in heavily polluted cities. Improved resolution in emission inventories will enable future simulations to better validate the ISF mechanism and more accurately capture the observed characteristics of heavily polluted sites like Wuhan. Additionally, comparisons with observations showed that simulated sulfate, nitrate, and ammonium concentrations under ISF were in a little more reasonable agreement with the observations than those of APF (Figure 1c,e, and Supporting Information Text S4). This improved agreement can be attributed, in part, to the spatial distribution changes resulting from the different formation mechanisms (Supporting Information Text S5), while APF and ISF simulate similar SO_2_ concentrations (Supporting Information Figure S5). Most importantly, the inclusion of ISF in the model resulted in a remarkable agreement with the observations, demonstrating a simulated accumulation mode with a modal size of 0.92 μm (0.68–0.92 μm, Figure 2 and Supporting Information Figure S3). This outcome signifies that ISF not only enhanced the accuracy of predicted sulfate mass concentrations but also provided a comprehensive explanation for the observed aerosol particle size distributions. The main difference between the APF and ISF mechanisms lies in the total processing volume. In the ISF mechanism, the processing volume is limited to fresh emission plumes, while the APF mechanism encompasses the entire boundary layer. Therefore, the APF processing volume is orders of magnitude larger than that of ISF. While the total supply of sulfate production is on the same order for the two mechanisms, the much smaller ISF processing volume means that the number of pre-existing particles onto which H_2_SO_4_ condenses is much smaller than those of APF. The much smaller number of ISF particles can therefore grow to much larger sizes than APF particles.

Two sensitivity experiments were conducted to examine ISF source parameters using the WRF-Chem model. In the first simulation (S1), we assumed that 50% of the sulfate was from the ISF source, and the other 50% was from the APF and CSF sources. The average modal sizes at the 12 observation sites (Supporting Information Figure S6) were 0.62 ± 0.09 μm, significantly lower than the observations (Supporting Information Figure S3). In the second simulation (S2), we increased the modal size of the ISF source from 0.9 to 2 μm while keeping its source at 50% of sulfate. The large difference in the size distributions of the APF and ISF sources resulted in two modal distributions peaking at 0.35 and 2 μm, respectively, whereas the observations have only one peak in the accumulation mode in most cases (Supporting Information Figure S6). Although the sulfate mass concentrations were close to the ISF simulations in both S1 and S2 simulations, the simulations cannot reproduce the observed size distributions. The observations also showed that the wintertime size distributions of nitrate and ammonium were generally similar to those of sulfate (Supporting Information Figure S7). Taken together, the observed size distributions of sulfate, nitrate, and ammonium and the sensitivity experiments indicated that the observed distributions provide an important constraint on the sources of sulfate, which is the observation basis for the ISF source being the integral source of sulfate in the winter in eastern China.

A recent study found that the sulfate formation rate of dissolved-manganese (Mn) catalyzed oxidation of SO_2_ on the aerosol surface was orders of magnitude faster than previously known aqueous reaction pathways.^13^ We speculate that the highly acidic and high-temperature environment of ISF may promote the rapid production of soluble metal and the catalytic oxidation of SO_2_. The initial ISF conversion of SO_3_ to sulfuric acid in aerosols provides an acidic environment to promote the rapid production of soluble transition metals, such as Mn(II). The ISF high-temperature can greatly increase the Mn(II)-catalyzed oxidation of SO_2_ to sulfuric acid since a 10 K increase from 278 K can speed up the aerosol-surface reaction by up to a factor of 10.^13^ The observations of similar size distributions between sulfate and Mn aerosols with a modal size of ∼1 μm^13^ are also consistent with this process. Depending on the efficiency of the ISF metal-catalyzed oxidation of SO_2_ on the aerosol surface or in aerosols, ISF sulfate formation may require a much smaller fraction of SO_3_ emissions than we assumed in this study. The sulfate production rate at high temperatures of exhaust plumes is currently unknown. Future studies are needed to account for this ISF oxidation pathway of SO_2_ based on experimental data as well as the variations in metal and SO_3_ emission rates from different combustion sources in order to quantify the contributions by SO_3_ conversion and heterogeneous SO_2_ oxidation to the ISF sulfate source.

### Impacts on Radiative Forcing and Human Health

**Figure 4:**
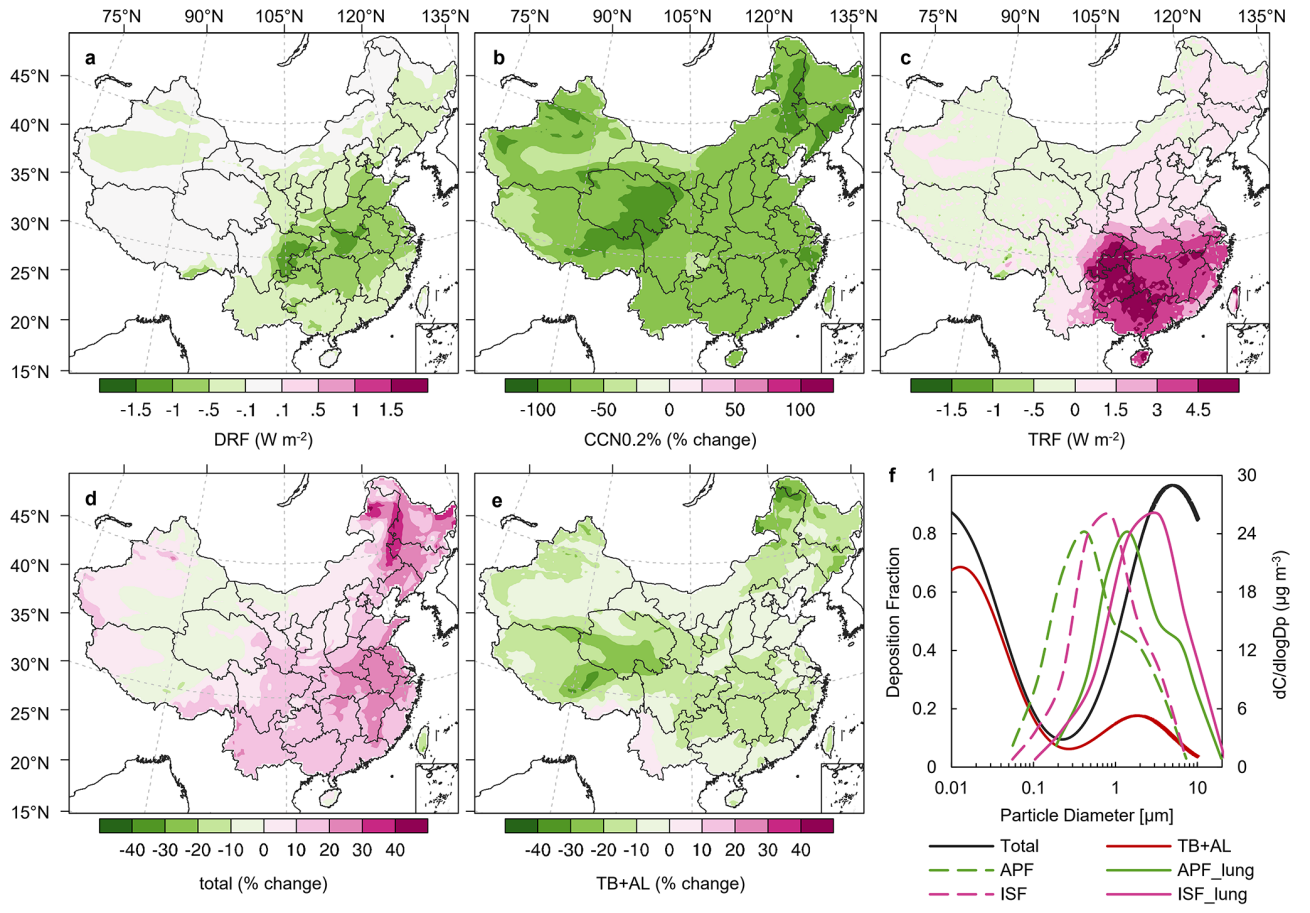
Impact of ISF on regional radiative forcing and human health assessments. (a–e) Changes from the APF to ISF simulation. (a) Changes in direct radiative forcing (DRF) at the surface. (b) Changes in CCN concentrations at a supersaturation of 0.2% (CCN0.2%) in the boundary layer (0–1 km). (c) Changes in total radiative forcing (TRF) at the surface. (d,e) Changes in deposited particle mass in the entire respiratory system (total) and tracheobronchial and alveolar regions (TB + AL) in humans. (f) Deposition fraction curves for particle deposition in different regions of the human respiratory system based on the International Commission on Radiological Protection (ICRP) model^70^ (solid red line and black line) and the particulate mass distribution at ambient wet diameters (dashed green line and pink line), at wet diameters for the relative humidity of the lung (solid green line and pink line), averaged over the North China Plain (Supporting Information Figure S1) for APF and ISF simulations.

Previous model simulations using standard regional models were unable to accurately simulate micron-sized particles. Consequently, previous evaluations of aerosol radiative and health impacts may not accurately reflect the actual influence of micrometer-sized particles observed in China. To emphasize the significance of considering aerosol size distributions when assessing the impacts of inorganic aerosols on radiative forcing and human health, we conducted an examination of the effects of APF and ISF sulfate sources on the evaluations of aerosol climate and public health impacts across various particle size distributions. We found that APF presumably underestimates the impact of aerosols on surface radiative heating, overestimates the deposition of particles in the tracheobronchial and alveolar regions, and underestimates the deposition of particles in the entire respiratory system. The total sulfate source was the same in both the APF and the ISF simulations. Although the APF and ISF simulated mass concentrations were more similar to each other than these two simulations were to the CSF simulations, the size distributions of the aerosols were substantially different (Figure 2). We used the ISF simulation and prescribed the APF aerosol distributions at each time step to examine the change in the direct and indirect surface radiative forcings between the two simulations. Figure 4a shows that the large inorganic aerosol distributions in the ISF increased surface radiative heating by 1.1 W m^–2^ over eastern China due in part to a decrease in the total aerosol scattering cross section and an increase in the asymmetry factor compared to the APF simulation.^85^ The largest ISF effects were found in Central China (1.7 W m^–2^) and Southwest China (1.9 W m^–2^), primarily due to high anthropogenic emissions and the abundance of solar radiation.

Furthermore, the aerosol size increase from the APF to ISF simulation decreased the number density of inorganic aerosols and, hence, the CCN. Using a supersaturation level of 0.2%,^69^ we found that ISF induced a 64% reduction in the CCN (Figure 4b). The number of particles acting as CCN influences the formation of cloud droplets and radiative characteristics of clouds, thereby affecting the aerosol indirect radiative forcing.^86^ The decrease in CCN further reduced the aerosol surface radiative forcing, increasing the surface heating from 1.1 to 2.7 W m^–2^ (Figure 4c). A larger effect was found in southern China than in northern China because the solar radiation, relative humidity, and cloud frequency are higher in southern China. Compared to −5 to 40 W m^–2^ of the anthropogenic aerosol effect on surface radiation in winter in eastern China assessed by other studies,^87^ the APF may underestimate 10% of the surface radiative effect in eastern China in winter (Figure 4c). Our results imply that some of the aerosol radiative cooling feedback to increased pollutants’ surface concentrations were overestimated in previous studies because of an underestimation of inorganic aerosol sizes.^88,89^

The change in the size of inorganic aerosols from the APF to ISF simulation also significantly alters the assessment results for public health. The respiratory deposition model showed that the deposition of aerosols into the respiratory system peaks for small (<0.01 μm) and coarse (>5 μm) particles (Figure 4f). The increase in aerosol size therefore increased the deposition of large particles (Figure 4d) but decreased that of small particles (Figure 4e). As a result, aerosol deposition in the entire respiratory system increased by 15% (10%–30%) over eastern China due to the increased deposition of large particles (Figure 4d), while the deposition of small particles in the bronchus and alveoli decreased by 10% (Figure 4e). The total aerosol deposition increase from the APF to ISF simulation varied significantly among the different regions, and the high population density regions tended to change more (Figure 4d,e, Supporting Information Figures S1 and S8). For example, because of the change from APF to ISF, the simulated increase in total deposition over the North China Plain, Sichuan Basin, Yangtze River Delta, and Pearl River Delta reached 12% to 22%, while a minor decrease occurred over sparsely populated western China. Thus, we argue that inorganic aerosols may have a greater health impact on the entire respiratory system and a smaller health effect on the bronchus and alveoli than previously thought.

### Implications for Air Pollution Control and Impact Assessments

A much greater focus has been placed on the formation of wintertime sulfate masses in China^9−11^ because sulfate aerosols are strongly related to climate change and human health impacts.^4,5,8^ We show in this work that the observations of aerosol size distributions with a modal size of 0.6–1.4 μm in China^29−32^ are much higher than that of 0.4–0.8 μm in North America.^25−28^ We find that APF and ISF mechanisms can simulate similar sulfate mass concentrations that are consistent with observations. However, their impact on the sulfate particle size distribution differs. Specifically, only the ISF mechanism can explain both the mass concentrations and the size distributions of sulfate. Our multiple lines of evidence demonstrate that ISF is most likely the dominant source of micrometer-sized sulfate particles in winter in China. There are several pathways of ISF through the condensation of existing ambient small aerosols of sulfuric acid produced by SO_3_ in fresh exhausts^36−39^ or rapid high-temperature catalytic SO_2_ oxidation by transition metals^13^ on acidic aerosols from the conversion of SO_3_ to sulfuric acid or the acid surface of the smokestack from the deposition of sulfuric acid. ISF may also explain the observed large sulfate particles with a modal size of 0.8–1.5 μm in other countries such as India, Vietnam, and Singapore.^90−93^ An increasing number of studies have shown that SO_3_ is important for air quality.^64,94^ Our findings further reinforce the critical role of sulfuric acid produced from the combustion emissions of SO_3_ in the formation of large sulfate particles. We suggest that policymakers pay more attention to controlling the direct emissions of SO_3_ and sulfate formation in the plumes. In the future, considering expected changes in emission sources due to the air pollution control policies, changes in the size distribution of particles may also occur due to modifications in emission sources and their associated particle size characteristics. Accurately assessing the effects of inorganic aerosols on radiative forcing and human health relies heavily on understanding the size distribution of aerosols. Thus, continuous monitoring and assessments are essential to evaluate the long-term impact of air pollution control measures on ISF.

On a broader scale, our research reveals that the ISF source of large sulfate aerosols has substantial implications. These aerosols contribute to increased surface heating and alter aerosol deposition in the human respiratory system, thereby influencing our understanding of the overall impacts of aerosols. That is, we show that an underestimation of inorganic aerosol sizes could lead to an overestimation of the aerosol radiative cooling feedback to increase the surface concentrations. Thus, our study is helpful in improving sulfate simulations in China by using atmospheric and climate models. Particle size plays a crucial role in the deposition of particulate matter in the human body. However, it is often overlooked in many health risk assessments, where the effects of different particle sizes on human health are implicitly disregarded. Keeping the mass concentration constant, ISF can lead to a higher total mass of larger particles deposited throughout the entire respiratory system. However, larger particles have a weaker ability to penetrate deep into the respiratory system, resulting in a smaller total mass deposition in the tracheobronchial and alveolar regions. Consequently, the health impact of larger particles on the tracheobronchial and alveolar regions is smaller. Another implication of ISF is that it may change our understanding of the biogeochemical cycle of elements such as sulfur and nitrogen because the particle size is a key factor that impacts atmospheric sulfate and nitrate deposition, which greatly affects soil pH, water quality, and greenhouse gas emissions.^95,96^ In conclusion, our findings suggest that future models should prioritize refining the processes involved in sulfate formation and incorporating the influence of the particle size. By doing so, we can effectively reduce uncertainties in climate change simulations and improve the accuracy of human health assessments.

## Supporting Information Available

Details of the configuration and test of the MOSAIC aerosol model, detailed description of the calculation of β in APF parametrization, importance of aerosol number size distribution comparison and current challenges, detailed evaluation of the simulations, and distributions of APF and ISF simulated sulfate, simulation domain of this study, the configuration of the ICRP model, the observed and model-simulated modal size of the accumulation mode for sulfate, aerosol surface area and volume distributions under the APF simulation, comparison of observed and simulated monthly average concentrations of SO_2_ and PM_2.5_, sulfate mass size distributions under different mechanisms, observed wintertime sulfate, nitrate, and ammonium mass size distributions, particulate deposition changes of eastern China, sulfate mass size distributions at background sites or unpolluted remote sites over China, spatial distributions of simulated mean concentrations of sulfate, nitrate, ammonium, and PM_2.5_ under different mechanisms, particle size range in the MOSAIC model, model configurations of the WRF-Chem model, summary of the observations, and statistics of meteorological variables. (PDF)
