# Supplementary material for: Wintertime Formation of Large Sulfate Particles in China and Implications for Human Health
Source: Environ Sci Technol. 2023 Nov 1;57(48):20010–23. doi: 10.1021/acs.est.3c05645 (PMC10702544; doi:10.1021/acs.est.3c05645)
Supplement: Supplementary file 1 — es3c05645_si_001.pdf [file es3c05645_si_001.pdf]

Supporting Information

Wintertime formation of large sulfate particles in China and implications for human health

Qianru Zhang[1,2], Yuhang Wang[2,*], Maodian Liu[1,3], Mingming Zheng[2,4], Lianxin Yuan[5], Junfeng Liu[1], Shu Tao[1], Xuejun Wang[1,*]

[1] Ministry of Education Laboratory of Earth Surface Processes, College of Urban and Environmental Sciences, Peking University, Beijing 100871, China

[2] School of Earth and Atmospheric Sciences, Georgia Institute of Technology, Atlanta, GA 30332, USA

[3] School of the Environment, Yale University, New Haven, CT 06511, USA

[4] School of Chemical and Environmental Engineering, Wuhan Polytechnic University, Wuhan, 430023, China

[5] Hubei Environmental Monitoring Center, Wuhan, 430072, China

*Corresponding authors:**

Yuhang Wang. School of Earth and Atmospheric Sciences, Georgia Institute of Technology, Atlanta, GA 30332, USA. Tel: +1 (404) 894-3995. Email: ywang@eas.gatech.edu

Xuejun Wang. Ministry of Education Laboratory of Earth Surface Processes, College of Urban and Environmental Sciences, Peking University, Beijing 100871, China. Tel: +86-10-62759190. Email: xjwang@urban.pku.edu.cn

Number of pages: 28

Number of supporting texts: 5

Number of supporting figures: 10

Number of supporting tables: 5

Supporting Texts

Supporting Text 1. Configuration and test of the MOSAIC aerosol model

The Model for Simulating Aerosol Interactions and Chemistry (MOSAIC) aerosol model can be executed using the modal or sectional approach[1]. In the WRF-Chem model, MOSAIC is implemented using the sectional framework and the model allows users to choose 4 or 8 discrete size bins to represent the aerosol size distributions. In this study, 8 bins were selected in the simulations (Supporting Table S1). MOSAIC includes the treatments of all major aerosol species, such as sulfate, nitrate, ammonium, elemental carbon, organic mass, sodium, chloride, calcium, water and other components. In MOSAIC, aerosol species are mixed internally in each bin, so the particles in a bin have the same chemical compositions. MOSAIC simulates both aerosol mass and number for each bin. MOSAIC includes the treatments of various chemical and microphysical processes of aerosols, including: nucleation, coagulation, condensation, thermodynamic equilibrium, phase equilibrium, and so on. For thermodynamic module, in MOSAIC, Multicomponent Taylor Expansion Method (MTEM)[2] is used to calculate the mean activity coefficients of electrolytes in the aqueous aerosols and Multicomponent Equilibrium Solver for Aerosols (MESA)[3] is used to solve the solid-liquid phase equilibrium. In addition, Adaptive Step Time-split Euler Method (ASTEM) is employed to deal with the gas-particle partitioning problem. It is coupled with the thermodynamic module MESA-MTEM to integrate the mass transfer equations for all size bins[1]. In MOSAIC, firstly, the particle growth or shrinkage induced by dynamic gas-particle partitioning of trace gases is estimated in a Lagrangian manner, and then, the transfer of particles between bins is determined by employing a two-moment method[1].

We compared the simulated sulfate mass size distribution with measurements at six background sites or unpolluted remote sites in China to reflect the capability of the model in terms of reproducing the size distributions of sulfate under general conditions (Supporting Fig. S9). Although the model-simulated accumulation-mode peaks of sulfate are slightly smaller than the observation, the model generally captures the size distribution of sulfate in the accumulation mode under general conditions. In this study, CSF pathways

lead to smaller sulfate size bins compared to the observations. We tested the simulated results for other components and found that WRF-Chem model also show similar biases of size distribution for nitrate and ammonium under CSF simulation. After introducing the ISF mechanism, the simulated size distribution of ammonium also significantly ameliorated. However, the simulated size distribution of nitrate does not change substantially, suggesting other mechanisms might controlling the size distribution of nitrate during winter hazes in China.

Supporting Text 2. Calculation of β in the APF simulation

The calculation method for β in the APF simulation is as follows. Firstly, we calculated the total sulfate contribution from the ISF in the ISF simulation for China. Then, we performed APF pre-simulations, where all settings are consistent with APF except for the value of β, which is set to 1. Subsequently, in the APF pre-simulation, we determined an appropriate parameter γ through a combination of previous literature and sensitivity analysis. The contribution of sulfate from the APF mechanism can be determined by computing the difference in sulfate mass before and after the APF process at each time step in the model. By summing up the monthly contributions of APF mechanism from all grid cells across different vertical layers within China in APF pre-simulation, we obtained the total APF contribution to sulfate for each specific month. Finally, the ratio of the total sulfate contributions from the ISF and APF processes in ISF simulation and APF pre-simulation is the value of β for each month. Upon obtaining the values of β, we incorporated them into the simulation of APF for each month, conducted additional simulations, and got the final APF results in the article.

Supporting Text 3. Aerosol number size distribution comparison and current challenges

In power plant plumes, the presence of gaseous H_2SO_4 can play an important role in the generation of new particles. Previous studies conducted in North America have suggested that H_2SO_4 generated from power plant precursor emissions can lead to new particle formation, particularly in cleaner background conditions[4]. However, it's important to consider the background aerosol conditions, as pre-existing particles in the ambient environment act as condensation sinks, hindering the formation of new particles[4]. Consequently, regions with cleaner conditions are more conducive to new particle formation. Our study focused specifically on the heavily polluted winter seasons in China, where the presence of a substantial number of pre-existing particles in the ambient air poses challenges for new particle formation. Recent observational data collected during China's winter season has revealed that particles within power plant plumes tend to exhibit larger sizes compared to background particles in the atmosphere[5]. As a result, we propose that H_2SO_4 from power plants in China during winter primarily contributes to the condensation and growth of larger particles.

Comparing observed and simulated particle number concentrations is of importance. Nevertheless, this comparison currently encounters two significant challenges. Firstly, the default particle size bins in the current model are relatively coarse for accurately comparing with observed number concentrations. New particle formation typically occurs below 0.05 μm, necessitating the inclusion of multiple detailed size bins below this threshold in the model to enable precise simulations. However, the WRF-Chem model with the MOSAIC aerosol mechanism, by default, divides aerosol sizes into only 8 bins. The first two size bins are 0.039-0.078 μm and 0.078-0.156 μm. The current model includes only one particle size bin for all particles with a diameter < 0.05 μm, which is insufficient for accurately simulating new particle formation and comparing it with observations. Secondly, there is currently a scarcity of high-precision and multivariate observational data available for power plant and other combustion plumes and their surrounding areas in China, which

limits our ability to compare with observed data. Acquiring such data is crucial for enhancing our understanding of this mechanism.

Supporting Text 4. Evaluation of model-simulated wintertime air pollutants

The CSF simulation provided reasonable results for meteorological variables over China in the winter (Supporting Table S5). At the Wuhan site, the CSF simulations of SO_2 (normalized bias +87.1%) and nitrate (+33.0%) were overestimated, and the CSF simulations of ammonium (-24.7%) were underestimated. The APF and ISF simulations showed that the overestimation of SO_2 improved from +87.1% (CSF) to +65.1% (APF) and +59.4% (ISF). We noticed that the normalized bias of SO_2 in the APF and ISF simulations was still large, which may be primarily caused by the uncertainty of emission heights and emission inventory of SO_2. Previous studies have suggested that the near-surface concentrations of SO_2 simulated by atmospheric chemistry models are sensitive to vertical profiles of emissions[6-8]. Here, we conducted a set of sensitivity analyses on anthropogenic emission heights and found that simulations using current sector-specific emission vertical profiles could enable well-predicted SO_2 concentrations at most stations in eastern China (Supporting Fig. S5). Thus, the current emission heights employed in the model were relatively reasonable on a large scale. Owing to differences in economic development and industrial structures, vertical emission profiles may differ among regions. The application of unified emission heights in the model may lead to uncertainties in the simulation results. In addition, the SO_2 emission inventory also has uncertainties that may affect the simulated SO_2 concentration near the ground at the Wuhan site.

For other pollutants at the Wuhan site, the simulation results showed that the overestimation of nitrate improved from +33.0% (CSF) to +0.3% (APF) and -1.0% (ISF), and the underestimation of ammonium improved from -24.7% (CSF) to -15.4% (APF) and -14.4% (ISF) (Figs. 1a, 1b, and 1c in the main text). The predicted nitrate and ammonium

concentrations at the 25 stations were also improved under APF and ISF (Fig. 1e). The normalized bias of the predicted nitrate levels improved from +54.9% (CSF) to +24.2% (APF) and +18.5% (ISF). The normalized bias of the predicted ammonium improved from -20.5% (CSF) to -5.1% (APF) and +4.1% (ISF). The concentrations of SO_2 and $PM_{2.5}$ at the other stations in eastern China were reasonably well predicted in the APF and ISF simulations (Supporting Fig. S5).

Supporting Text 5. Distributions of APF and ISF simulated sulfate

Although the APF or ISF mechanism can greatly improve the simulated inorganic aerosol mass concentrations, and the total APF and ISF sources of sulfate are almost the same (see Methods), the spatial distributions of sulfate are substantially different (Supporting Fig. S10). For example, the surface concentration of ISF sulfate was higher over the North China Plain (ISF: 12.9 μg m^{-3} vs. APF: 7.8 μg m^{-3}), but lower over the Sichuan Basin (ISF: 18.8 μg m^{-3} vs. APF: 22.4 μg m^{-3}). This is because these two mechanisms are affected by different factors. The ISF mechanism is mainly affected by the sulfur emission distribution, whereas the APF mechanism is affected by not only SO_2 emissions but also the spatial distribution of the aerosol surface area. The higher aerosol surface concentrations over the Sichuan Basin than over the North China Plain lead to higher sulfate results over the Sichuan Basin in the APF simulation. Overall, Fig. 1 in the main text shows that the ISF-simulated sulfate concentrations were better than the APF results.

Supporting Figures

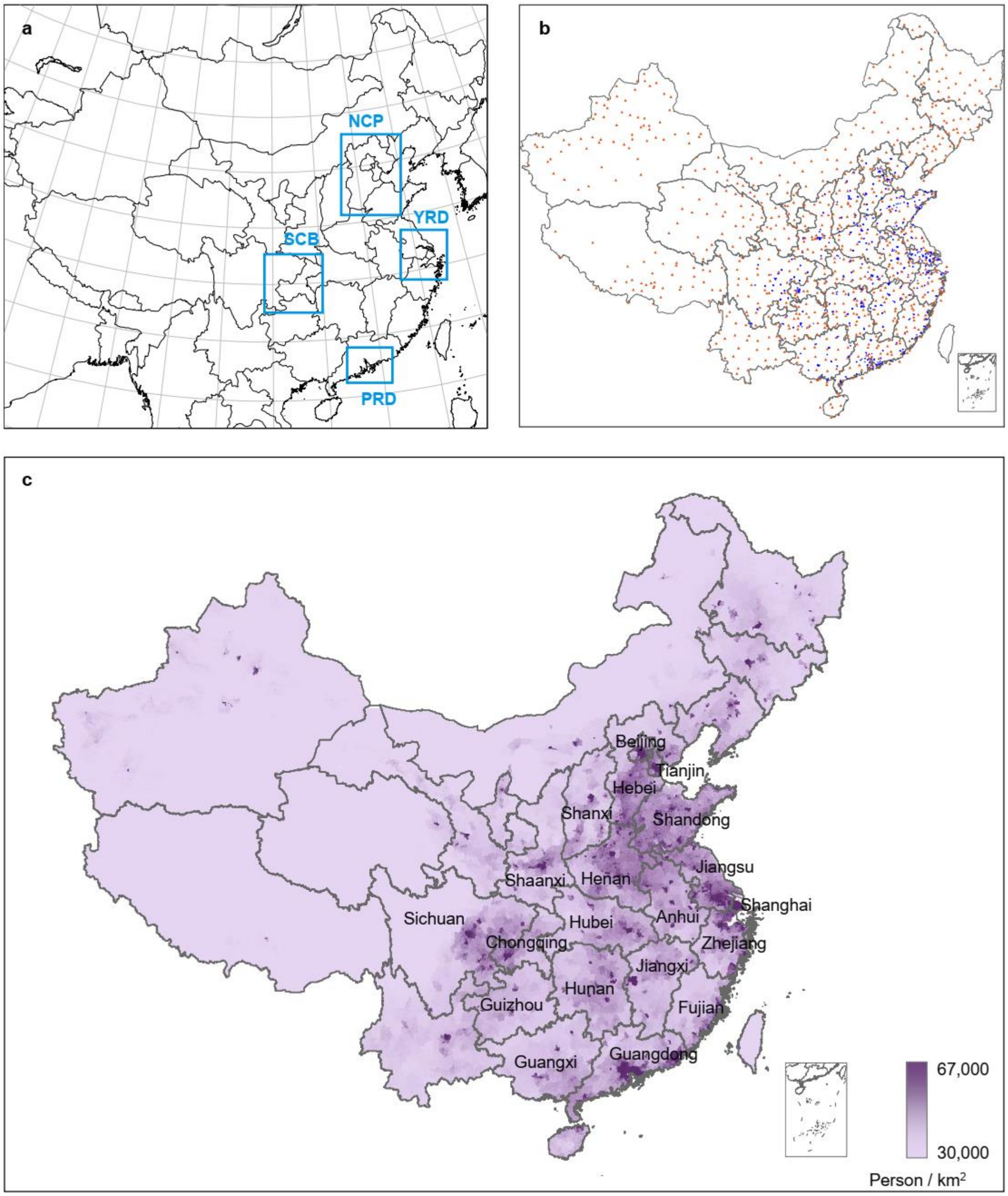

Supporting Fig. S1 Panel **a**. Model domain of the WRF-Chem simulation. The area in the blue boxes are the North China Plain (NCP), the Sichuan Basin (SCB), the Yangtze River Delta (YRD), and the Pearl River Delta (PRD) region used in this study. Panel **b**. Spatial distribution of the air quality stations (blue dots) and weather monitoring stations (orange

triangles) used in this study. Panel **c**. Location of the provinces in eastern China. The background is the spatial distribution of population density in 2015[9].

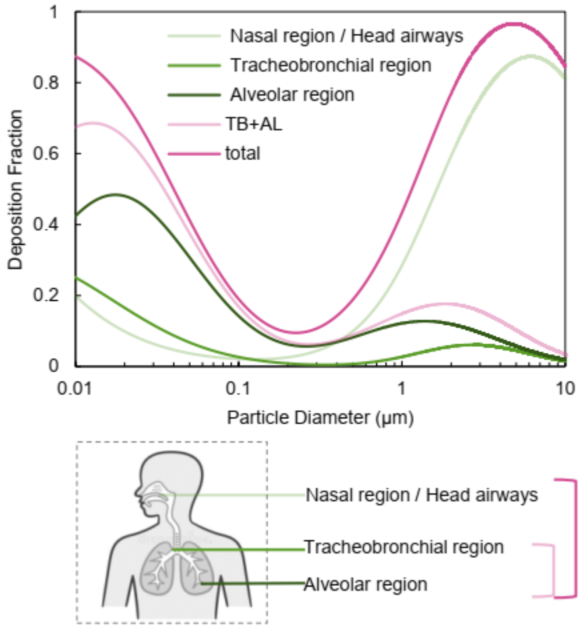

Supporting Fig. S2 Particulate mass deposition fraction curves in different regions of the human respiratory system based on ICRP model[10]. Lines ranging from light green to dark green represent different regions of the respiratory tract: nasal region /head airways, tracheobronchial region, and alveolar region. The light pink line (TB+AL) represents the sum of the tracheobronchial and alveolar regions, and the dark pink line (total) represents the sum of all three respiratory regions.

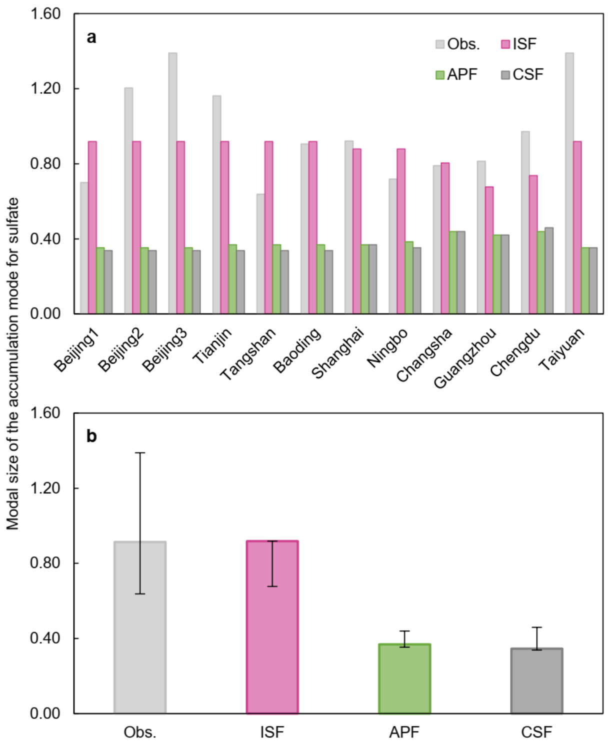

Supporting Fig. S3 Observed and model-simulated modal size of the accumulation mode for sulfate at 12 sites in China from December 2014 to February 2015. "Obs." denotes the observations[11-22].

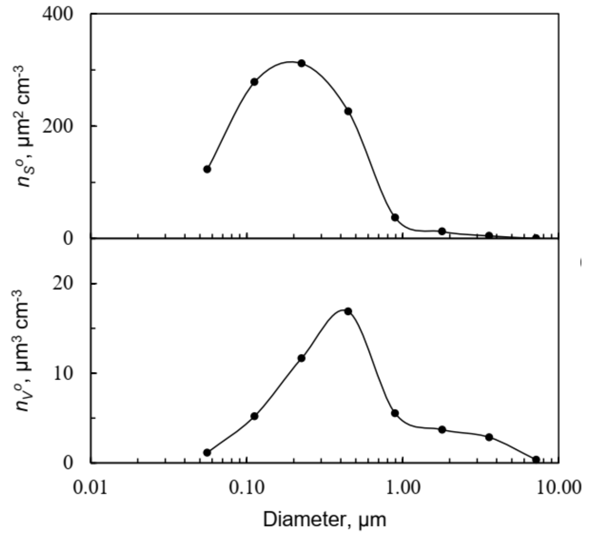

Supporting Fig. S4 Aerosol surface area (n_S^o) and volume (n_V^o) distributions averaged over the North China Plain under the APF simulation.

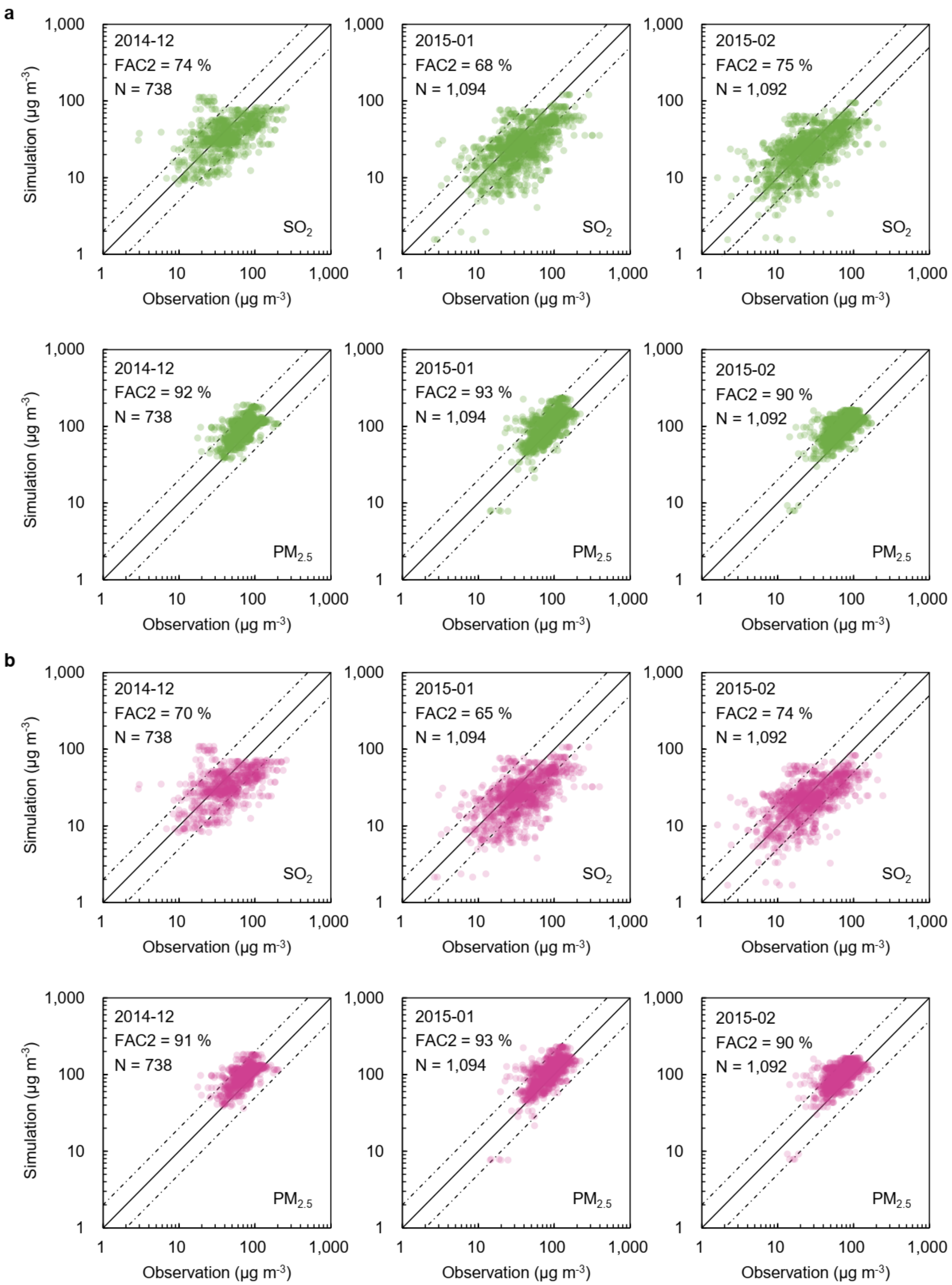

Supporting Fig. S5 Comparison between observed and simulated monthly average concentrations of SO_2 and $PM_{2.5}$ at air quality stations in China from December 2014 to

February 2015 under APF (panel **a)** and ISF (panel **b**). Fraction of model results within a factor of two of the observations (FAC2) and the number of stations (N) are displayed on each subplot.

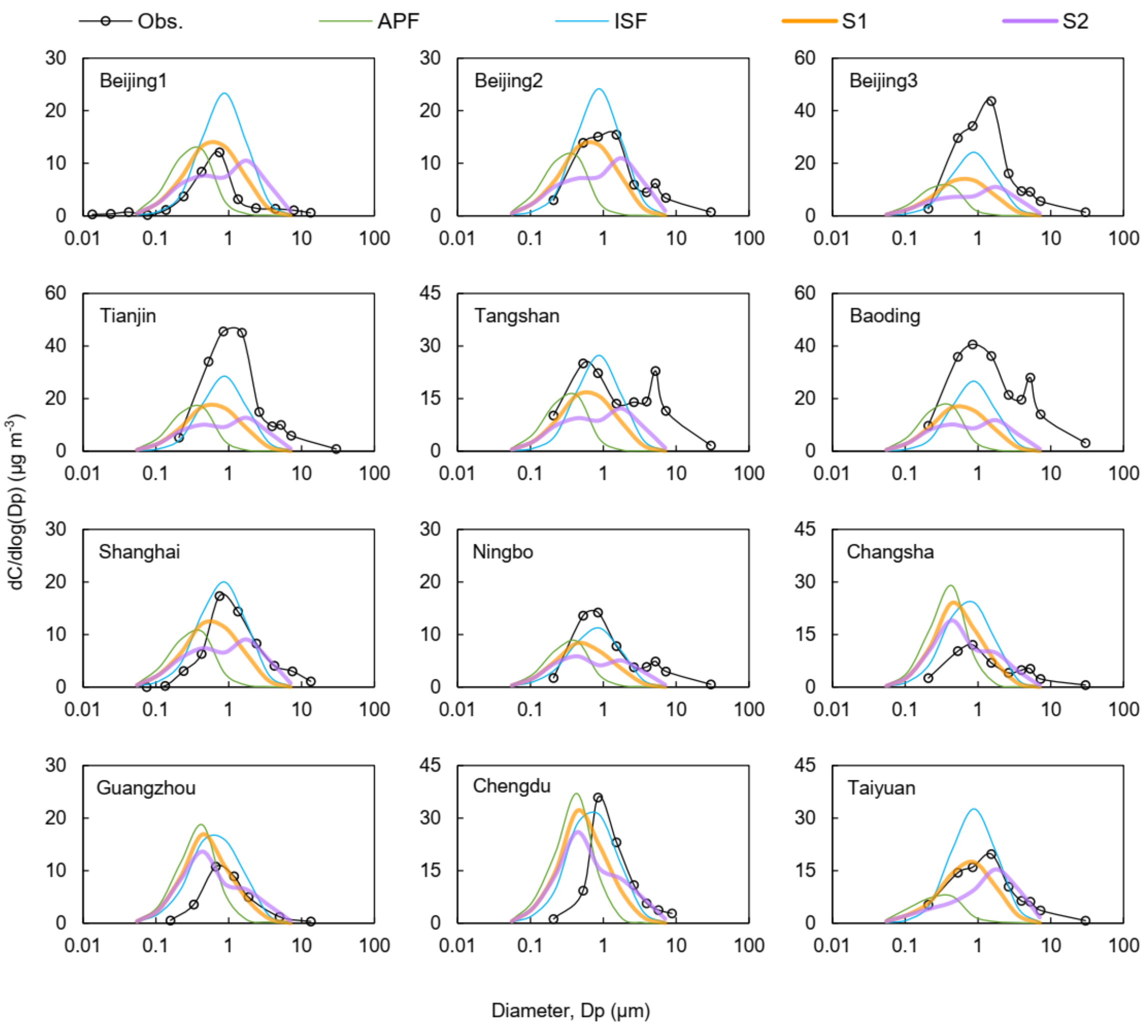

Supporting Fig. S6 Observed and simulated sulfate mass size distributions at 12 sites over China in winter. If the observation time of one site is within the simulation period, the simulated mean value of the data during exactly observation time was used to compare with the observation data (obs.) (listed in Supporting Table S4); otherwise, the three-month average values were employed to compare with the observation data. APF, ISF, S1, S2 represent different sulfate formation mechanisms (see the main text for details).

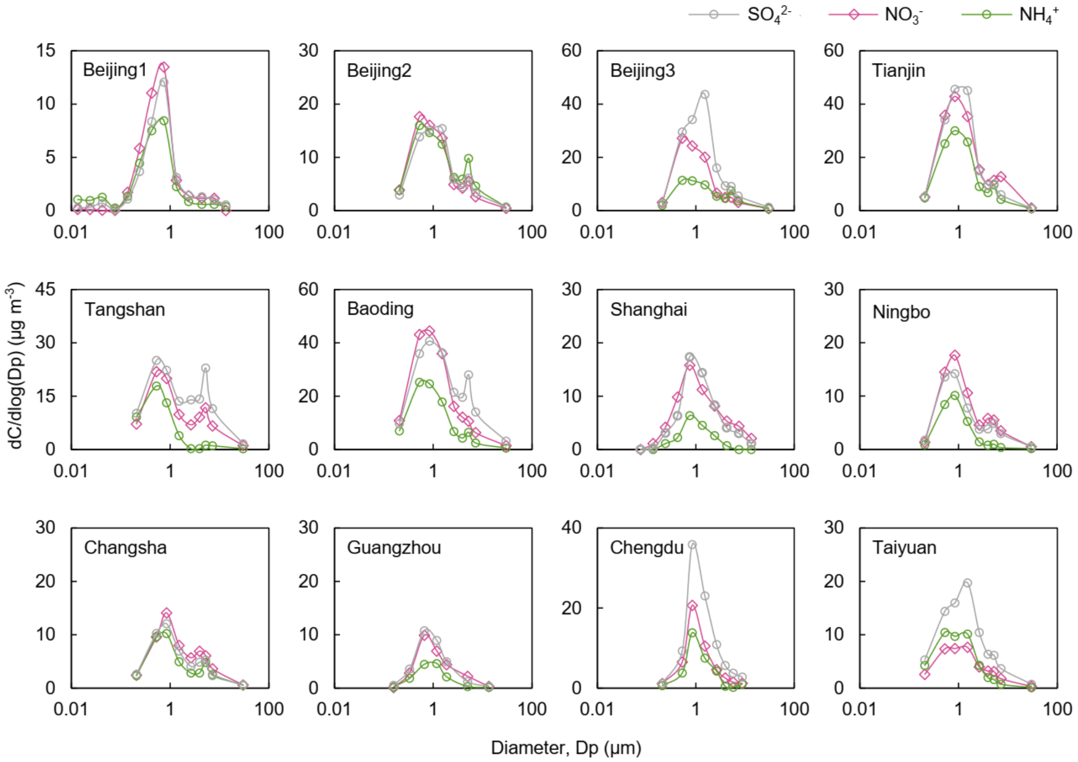

Supporting Fig. S7 Observed wintertime sulfate, nitrate, and ammonium mass size distributions at 12 sites in China.

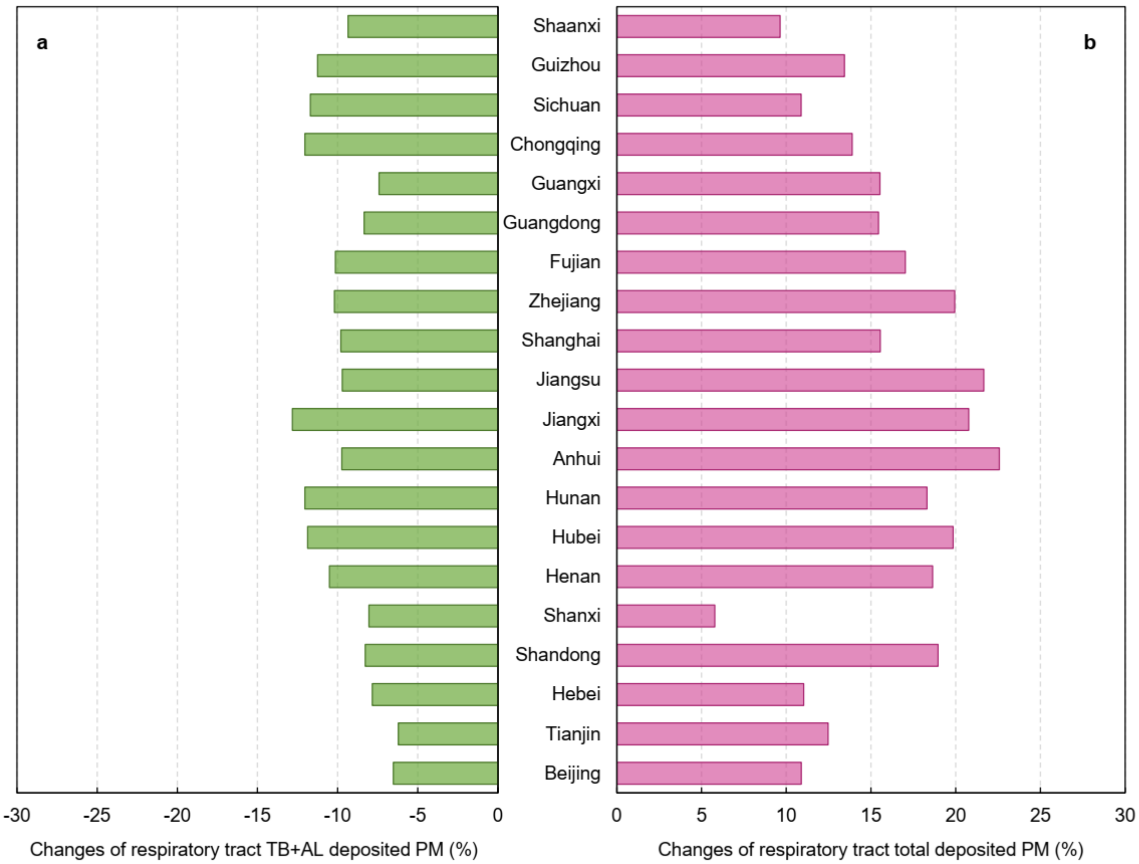

Supporting Fig. S8 Percentage particulate deposition changes of respiratory tract tracheobronchial and alveolar regions (TB+AL) and the total respiratory system of eastern China. The location of each province is shown in Supporting Fig. S1.

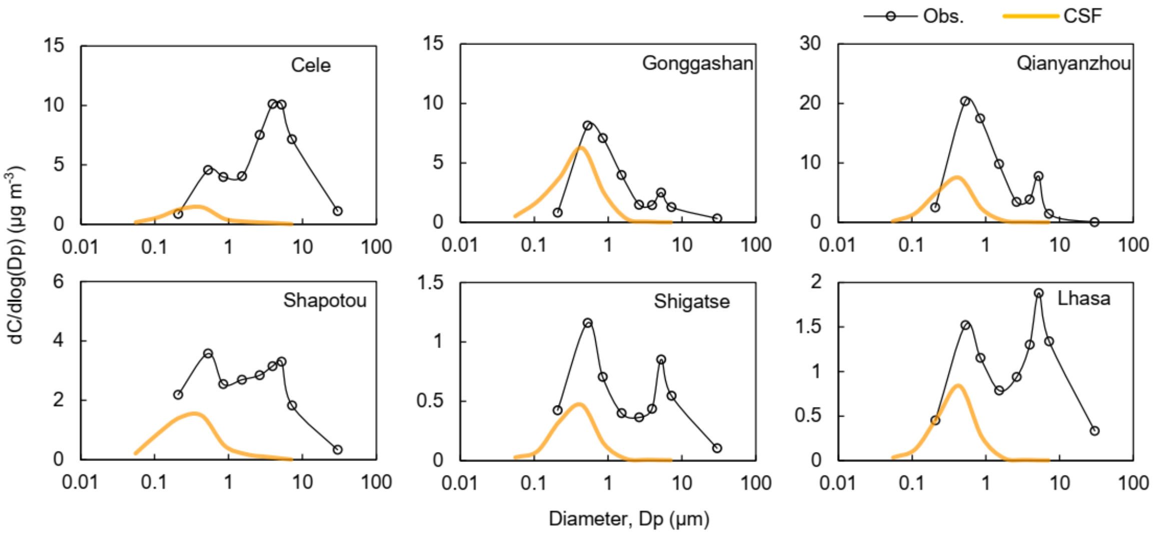

Supporting Fig. S9 Observed and simulated sulfate mass size distributions at six background sites or unpolluted remote sites over China in winter. The three-month average values were employed to compare with the observation data (Obs.), which was obtained from literature[23-25] and sampled between 2012-2014. CSF represents the classical sulfate formation mechanism.

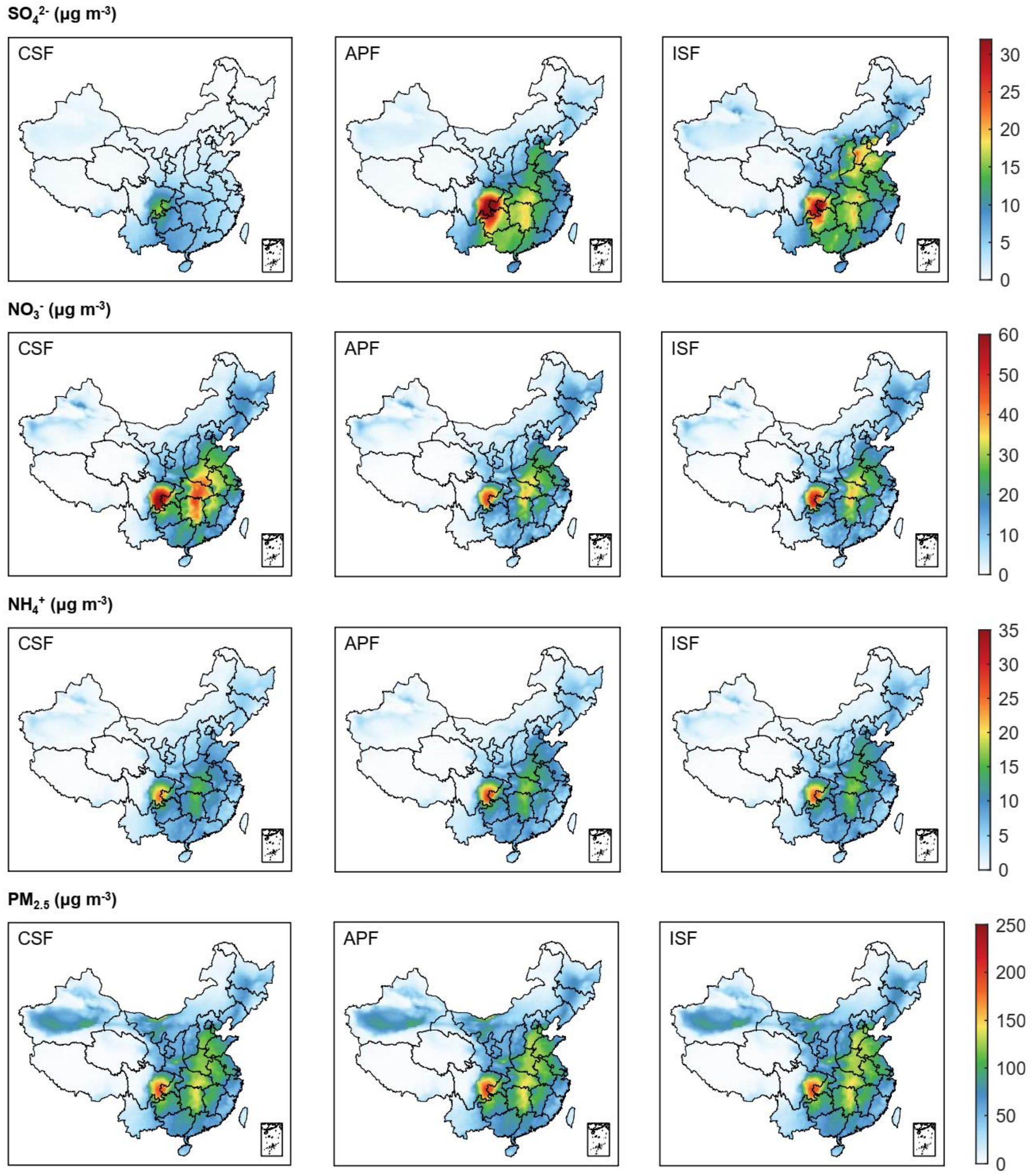

Supporting Fig. S10 Spatial distributions of simulated mean concentrations of sulfate, nitrate, ammonium, and $PM_{2.5}$ from December 2014 to February 2015 under CSF, APF, ISF.

Supporting Tables

Supporting Table S1. Particle size range of the eight bins in the MOSAIC.

Bin	Particle dry diameter (μm)
One	0.0390625-0.078125
Two	0.078125-0.15625
Three	0.15625-0.3125
Four	0.3125-0.625
Five	0.625-1.25
Six	1.25-2.5
Seven	2.5-5.0
Eight	5.0-10.0

Supporting Table S2. Main model configurations of the WRF-Chem model.

Items	Description
Model version	Version 3.9
Simulated time	Dec. 2014, Jan. 2015, Feb. 2015
Horizontal resolution	30 km × 30 km
Vertical resolution	30 vertical levels with model top at 50 hPa
Gas-phase chemical mechanism	CBMZ scheme[26]
Aerosol scheme	Eight-bin MOSAIC scheme[1]
Photolysis scheme	FAST-J photolysis scheme[27]
Cloud microphysics	Lin et al. scheme [28]
Shortwave radiation	RRTMG scheme [29]
Longwave radiation	RRTMG scheme [29]
Cumulus physics	Grell-3 scheme [30]
Land surface scheme	unified Noah land-surface model[31]
Land-use data	MODIS IGBP 21-category data
Surface layer scheme	Revised MM5 Monin-Obukhov scheme (Jimenez, renamed in v3.6)[32, 33]
Boundary layer scheme	YSU (Hong et al., 2006)[34]
Meteorological conditions	NCEP 1 × 1degree Final Analyses (FNL) data
Chemical initial and boundary conditions	CAM-Chem 6-h output data
Spin-up time	Five days

Supporting Table S3. Summary of the sulfate, nitrate, ammonium mass concentration observations.

No.	City	Longitude	Latitude	Period of observation	References
1	Beijing	116.32	39.99	2015.1	Zhang et al., 2019[35]
2	Beijing	116.32	39.99	2015.2	Zhang et al., 2019[35]
3	Beijing	116.38	39.97	2014.12.29 - 2015.1.27	Huang et al., 2017[36]
4	Chengdu	104.07	30.62	2015.1.6 - 2015.2.2	Wang et al., 2018[37]
5	Chengdu	104.68	30.63	2015.1	Kong et al., 2020[38]
6	Chengdu	104.68	30.63	2015.2	Kong et al., 2020[38]
7	Chongqing	106.51	29.62	2015.1.6 - 2015.2.2	Zhang et al., 2019[35]
8	Guangzhou	113.35	23.12	2014.12.24 - 2015.1.24	Tao et al., 2017[39]
9	Haikou	110.34	20.00	2015.1	Zhang et al., 2019[35]
10	Handan	114.51	36.61	2015.1	Ma et al., 2017[40]
11	Hefei	117.24	31.85	2014.12	Zhang et al., 2019[35]
12	Lin’an	119.73	30.30	2014.12.23 - 2015.1.22	Du et al., 2017[41]
13	Luoyang	112.26	34.40	2015.1. 22 - 2015.2.6	Jiang et al., 2017[42]
14	Nanjing	118.97	32.10	2014.12	Zhang et al., 2019[35]
15	Nanjing	118.97	32.10	2015.1	Zhang et al., 2019[35]
16	Nanjing	118.97	32.10	2015.2	Zhang et al., 2019[35]
17	Nanjing	118.78	32.05	2014.12.23 - 2015.1.22	Du et al., 2017[41]
18	Ningbo	121.53	29.86	2014.12.23 - 2015.1.22	Du et al., 2017[41]
19	Pingdingshan	113.19	33.43	2015.1.21 - 2015.2.5	Jiang et al., 2017[42]
20	Shanghai	121.39	31.17	2014.12.23 - 2015.1.22	Du et al., 2017[41]
21	Shenyang	123.44	41.79	2015.1 - 2015.2	Zhang et al., 2019[35]
22	Shijiazhuang	114.53	38.03	2014.12.29 - 2015.1.27	Huang et al., 2017[36]
23	Tianjin	117.19	39.09	2014.12.29 - 2015.1.27	Huang et al., 2017[36]
24	Xiamen	118.15	24.48	2014.12.10 - 2015.1.9	Zhang et al., 2016[43]
25	Xi'an	108.89	34.23	2014.12	Zhang et al., 2019[35]
26	Xi'an	108.89	34.23	2015.1	Zhang et al., 2019[35]
27	Xi'an	108.89	34.23	2015.2	Zhang et al., 2019[35]
28	Xinglong	117.58	40.39	2014.12.29 - 2015.1.27	Huang et al., 2017[36]

29	Zhengzhou	113.65	34.79	2015.1	Zhang et al., 2019[35]
30	Zhengzhou	113.32	34.50	2014.12.30 - 2015.1.15	Jiang et al., 2017[42]
31	Zhuhai	113.53	22.37	2014.12.24 - 2015.1.24	Tao et al., 2017[39]

Supporting Table S4. Summary of the sulfate, nitrate, ammonium size distribution observations.

No.	City	Lon	Lat	Altitude (m)	Station Type	Sampling period	Season	Sampling instrument	Number and duration of samples	Number of samples or sampling frequency	Reference
1	Beijing1	116.30	39.94	36	Urban	2017.1.14 - 2017.1.20	winter	MOUDI-122	14 × 11 hours	Number: 14	Du et al., 2018[11]
2	Beijing2	116.37	39.97	8	Urban	2014.1.8 - 2014.1.19	winter	Anderson Series 20-800, USA	11 × 23 hours	Number: 11	Huang et al., 2016[12]
3	Beijing3	116.42	39.98	40	Urban	2013.1.14 - 2013.1.27	winter	An eight-stage low-pressure impactor 20-800, Thermo Fisher Scientific, USA	NA × 24 hours	NA[1]	Yang et al., 2015[13]
4	Tianjin	117.20	39.07	2.2	Urban	2014.1.8 - 2014.1.19	winter	Anderson Series 20-800, USA	NA × 23 hours	NA	Yao et al., 2020[14]
5	Tangshan	118.20	39.60	14	Urban	2009-2010-winter	winter	Andersen Series 20-800, USA	NA × 24 hours	Biweekly	Li et al., 2013[15]
6	Baoding	115.40	38.80	NA	Urban	2010-2011-winter	winter	Andersen Series 20-800, USA	NA × 24 hours	Biweekly	Wang et al., 2013[16]
7	Shanghai	121.50	31.30	15	Urban	2016.1	winter	MOUDI, MSP Corp.,USA; Model 110-R	NA × 24 hours	NA	Ding et al., 2017[17]

8	Ningbo	121.90	29.75	20	Coastal suburb, several industries around the site	2014.12.24 - 2015.1.22	winter	Anderson Series 20–800, USA	8 × 3 days	Number: 8	Zhang et al., 2018[18]
9	Changsha	113.07	28.20	58	Urban	2014-winter	winter	Anderson impact grading sampler (Series 20-800 Mark II), American Thermoelectric Corporation	NA × 48 hours	Weekly	Wu et al., 2020[19]
10	Guangzhou	113.32	23.11	0	Urban	2015.12 - 2016.1	winter	Six-stage samplers (Model 131 High-Flow Impactor, MSP Corporation)	13 × 24 hours	Number: 13	Zhou et al., 2020[20]
11	Chengdu	104.13	30.67	15	Urban	2012-2013-winter	winter	Andersen cascade impactor air sampler (AN-200; SIBATA, Japan)	NA × 4-8 days	NA	Li et al., 2019[21]
12	Taiyuan	112.53	37.87	15	Urban	2012-2014-winters	winter	Andersen, Series 20-800, USA	NA × 48 hours	Biweekly	Wang et al., 2016[22]

[1] NA denotes that the data is not available.

Supporting Table S5. Statistics of meteorological variables in WRF-Chem at all sites over China.

Time	Meteorological variables	Mean observation	Mean simulation
2014-12	1.5m relative humidity (%)	58.7	56.3
	1.5m temperature (℃)	-0.6	-1.5
	10m wind speed (m/s)	2.1	3.2
2015-01	1.5m relative humidity (%)	63.3	61.7
	1.5m temperature (℃)	-0.1	-1.0
	10m wind speed (m/s)	2.0	2.9
2015-02	1.5m relative humidity (%)	62.0	61.4
	1.5m temperature (℃)	2.4	1.3
	10m wind speed (m/s)	2.2	3.1

References

1. Zaveri, R. A.; Easter, R. C.; Fast, J. D.; Peters, L. K., Model for Simulating Aerosol Interactions and Chemistry (MOSAIC). *J. Geophys. Res.-Atmos.* **2008,** *113*, (D13), D13204.
2. Zaveri, R. A.; Easter, R. C.; Wexler, A. S., A new method for multicomponent activity coefficients of electrolytes in aqueous atmospheric aerosols. *J. Geophys. Res.-Atmos.* **2005,** *110*, (D2).
3. Zaveri, R. A.; Easter, R. C.; Peters, L. K., A computationally efficient multicomponent equilibrium solver for aerosols (MESA). *J. Geophys. Res.-Atmos.* **2005,** *110*, (D24).
4. Stevens, R. G.; Pierce, J. R.; Brock, C. A.; Reed, M. K.; Crawford, J. H.; Holloway, J. S.; Ryerson, T. B.; Huey, L. G.; Nowak, J. B., Nucleation and growth of sulfate aerosol in coal-fired power plant plumes: sensitivity to background aerosol and meteorology. *Atmos Chem Phys* **2012,** *12*, (1), 189-206.
5. Shen, Y.; Meng, H.; Yao, X.; Peng, Z.; Sun, Y.; Zhang, J.; Gao, Y.; Feng, L.; Liu, X.; Gao, H., Does Ambient Secondary Conversion or the Prolonged Fast Conversion in Combustion Plumes Cause Severe PM2.5 Air Pollution in China? *Atmosphere-Basel* **2022,** *13*, (5), 673.
6. Yang, Y.; Smith, S. J.; Wang, H. L.; Lou, S. J.; Rasch, P. J., Impact of Anthropogenic Emission Injection Height Uncertainty on Global Sulfur Dioxide and Aerosol Distribution. *J. Geophys. Res.-Atmos.* **2019,** *124*, (8), 4812-4826.
7. Bieser, J.; Aulinge, A.; Matthias, V.; Quante, M.; van der Gon, H. A. C. D., Vertical emission profiles for Europe based on plume rise calculations. *Environ. Pollut.* **2011,** *159*, (10), 2935-2946.
8. Mailler, S.; Khvorostyanov, D.; Menut, L., Impact of the vertical emission profiles on background gas-phase pollution simulated from the EMEP emissions over Europe. *Atmos Chem Phys* **2013,** *13*, (12), 5987-5998.
9. Xu, X., China population spatial distribution kilometer grid dataset. *Data Registration and publishing System of Resources and Environmental Sciences Data Center, Chinese Academy of Sciences* **2017**.
10. Hinds, W. C., *Aerosol technology: properties, behavior, and measurement of airborne particles*. John Wiley & Sons: 1999.
11. Du, X.; Zhao, P.-S.; Su, J.; Dong, Q., Size distributions of water-soluble components in ambient aerosol of Beijing. *Environ. Sci.* **2018,** *39*, (11), 4858-4865 (in Chinese with English abstract).
12. Huang, X. J.; Liu, Z. R.; Zhang, J. K.; Wen, T. X.; Ji, D. S.; Wang, Y. S., Seasonal variation and secondary formation of size-segregated aerosol water-soluble inorganic ions during pollution episodes in Beijing. *Atmos. Res.* **2016,** *168*, 70-79.
13. Yang, Y.; Zhou, R.; Wu, J.; Yu, Y.; Ma, Z.; Zhang, L., Seasonal variations and size distributions of water-soluble ions in atmospheric aerosols in Beijing, 2012. *J. Environ. Sci.* **2015,** *34*, 197-205.
14. Yao, Q.; Liu, Z. R.; Han, S. Q.; Cai, Z. Y.; Liu, J. L.; Hao, T. Y.; Liu, J. Y.; Huang, X. J.; Wang, Y. S., Seasonal variation and secondary formation of size-segregated aerosol water-soluble inorganic ions in a coast megacity of North China Plain. *Environ Sci Pollut R* **2020,** *27*, (21), 26750-26762.
15. Li, X. R.; Wang, L. L.; Ji, D. S.; Wen, T. X.; Pan, Y. P.; Sun, Y.; Wang, Y. S., Characterization of the size-segregated water-soluble inorganic ions in the Jing-Jin-Ji urban agglomeration: Spatial/temporal variability, size distribution and sources. *Atmos Environ* **2013,** *77*, 250-259.
16. Wang, L.; Wen, T.-x.; Miao, H.-y.; Li, X.-r.; Xu, Z.-j.; Wang, Y.-s., Concentrations and Size Distributions of Water Soluble Inorganic Ion in Aerosol Particles in Baoding, Hebei. *Res. Environ. Sci.* **2013,** *26*, (5), 516-521 (in Chinese with English abstract).

17. Ding, X. X.; Kong, L. D.; Du, C. T.; Zhanzakova, A.; Fu, H. B.; Tang, X. F.; Wang, L.; Yang, X.; Chen, J. M.; Cheng, T. T., Characteristics of size-resolved atmospheric inorganic and carbonaceous aerosols in urban Shanghai. *Atmos Environ* **2017,** *167*, 625-641.
18. Zhang, J. J.; Tong, L.; Huang, Z. W.; Zhang, H. L.; He, M. M.; Dai, X. R.; Zheng, J.; Xiao, H., Seasonal variation and size distributions of water-soluble inorganic ions and carbonaceous aerosols at a coastal site in Ningbo, China. *Sci. Total Environ.* **2018,** *639*, 793-803.
19. Wu, X. Y.; Xin, J. Y.; Zhang, W. Y.; Gong, C. S.; Ma, Y. N.; Ma, Y. J.; Wen, T. X.; Liu, Z. R.; Tian, S. L.; Wang, Y. S.; Wu, F. K., Optical, Radiative and Chemical Characteristics of Aerosol in Changsha City, Central China. *Adv Atmos Sci* **2020,** *37*, (12), 1310-1322.
20. Zhou, S. Z.; Wu, L. L.; Guo, J. C.; Chen, W. H.; Wang, X. M.; Zhao, J.; Cheng, Y. F.; Huang, Z. Z.; Zhang, J. P.; Sun, Y. L.; Fu, P. Q.; Jia, S. G.; Tao, J.; Chen, Y. N.; Kuang, J. X., Measurement report: Vertical distribution of atmospheric particulate matter within the urban boundary layer in southern China - size-segregated chemical composition and secondary formation through cloud processing and heterogeneous reactions. *Atmos Chem Phys* **2020,** *20*, (11), 6435-6453.
21. Li, Q. K.; Yang, Z.; Li, X. D.; Ding, S. Y.; Du, F., Seasonal Characteristics of Sulfate and Nitrate in Size-segregated Particles in Ammonia-poor and -rich Atmospheres in Chengdu, Southwest China. *Aerosol Air Qual. Res.* **2019,** *19*, (12), 2697-2706.
22. Wang, L.; Wen, T.-X.; Miao, H.-Y.; Gao, W.-K.; Wang, Y.-S., Concentrations and size distributions of water-soluble inorganic ions in aerosol particles in Taiyuan, Shanxi. *Environ. Sci.* **2016,** *37*, (9), 3249-3257 (in Chinese with English abstract).
23. Wang, L. Characteristics and regional distributions of size-segregated water-soluble inorganic ions in atmospheric particulate matters in China. University of Chinese Academy of Sciences (in Chinese with English abstract), 2017.
24. Yang, Y.; Zhou, R.; Yan, Y.; Yu, Y.; Liu, J.; Du, Z.; Wu, D., Seasonal variations and size distributions of water-soluble ions of atmospheric particulate matter at Shigatse, Tibetan Plateau. *Chemosphere* **2016,** *145*, 560-567.
25. Wan, X.; Kang, S.; Xin, J.; Liu, B.; Wen, T.; Wang, P.; Wang, Y.; Cong, Z., Chemical composition of size-segregated aerosols in Lhasa city, Tibetan Plateau. *Atmos. Res.* **2016,** *174*, 142-150.
26. Zaveri, R. A.; Peters, L. K., A new lumped structure photochemical mechanism for large-scale applications. *J. Geophys. Res.-Atmos.* **1999,** *104*, (D23), 30387-30415.
27. Wild, O.; Zhu, X.; Prather, M. J., Fast-j: Accurate simulation of in- and below-cloud photolysis in tropospheric chemical models. *J. Atmos. Chem.* **2000,** *37*, (3), 245-282.
28. Lin, Y. L.; Farley, R. D.; Orville, H. D., Bulk Parameterization of the Snow Field in a Cloud Model. *J. Clim. Appl. Meteorol.* **1983,** *22*, (6), 1065-1092.
29. Iacono, M. J.; Delamere, J. S.; Mlawer, E. J.; Shephard, M. W.; Clough, S. A.; Collins, W. D., Radiative forcing by long-lived greenhouse gases: Calculations with the AER radiative transfer models. *J. Geophys. Res.-Atmos.* **2008,** *113*, (D13), D13103.
30. Grell, G. A.; Devenyi, D., A generalized approach to parameterizing convection combining ensemble and data assimilation techniques. *Geophys. Res. Lett.* **2002,** *29*, (14), 1693.
31. Tewari, M.; Chen, F.; Wang, W.; Dudhia, J.; LeMone, M.; Mitchell, K.; Ek, M.; Gayno, G.; Wegiel, J.; Cuenca, R., Implementation and verification of the unified NOAH land surface model in the WRF model. *20th conference on weather analysis and forecasting/16th conference on numerical weather prediction* **2004,** *11-15*, (6).
32. Monin, A. S.; Obukhov, A. M., Basic laws of turbulent mixing in the surface layer of the atmosphere. *Contrib. Geophys. Inst. Acad. Sci. USSR* **1954,** *151*, (163), 163–187.
33. Jimenez, P. A.; Dudhia, J.; Gonzalez-Rouco, J. F.; Navarro, J.; Montavez, J. P.; Garcia-Bustamante, E., A Revised Scheme for the WRF Surface Layer Formulation. *Mon. Weather Rev.* **2012,** *140*, (3), 898-918.

34. Hong, S. Y.; Noh, Y.; Dudhia, J., A new vertical diffusion package with an explicit treatment of entrainment processes. *Mon. Weather Rev.* **2006,** *134*, (9), 2318-2341.
35. Zhang, Q.; Zheng, Y. X.; Tong, D.; Shao, M.; Wang, S. X.; Zhang, Y. H.; Xu, X. D.; Wang, J. N.; He, H.; Liu, W. Q.; Ding, Y. H.; Lei, Y.; Li, J. H.; Wang, Z. F.; Zhang, X. Y.; Wang, Y. S.; Cheng, J.; Liu, Y.; Shi, Q. R.; Yan, L.; Geng, G. N.; Hong, C. P.; Li, M.; Liu, F.; Zheng, B.; Cao, J. J.; Ding, A. J.; Gao, J.; Fu, Q. Y.; Huo, J. T.; Liu, B. X.; Liu, Z. R.; Yang, F. M.; He, K. B.; Hao, J. M., Drivers of improved PM2.5 air quality in China from 2013 to 2017. *Proc. Natl. Acad. Sci. U. S. A.* **2019,** *116*, (49), 24463-24469.
36. Huang, X. J.; Liu, Z. R.; Liu, J. Y.; Hu, B.; Wen, T. X.; Tang, G. Q.; Zhang, J. K.; Wu, F. K.; Ji, D. S.; Wang, L. L.; Wang, Y. S., Chemical characterization and source identification of PM2.5 at multiple sites in the Beijing-Tianjin-Hebei region, China. *Atmos Chem Phys* **2017,** *17*, (21), 12941-12962.
37. Wang, H.; Tian, M.; Chen, Y.; Shi, G.; Liu, Y.; Yang, F.; Zhang, L.; Deng, L.; Yu, J.; Peng, C., Seasonal characteristics, formation mechanisms and source origins of PM 2.5 in two megacities in Sichuan Basin, China. *Atmos Chem Phys* **2018,** *18*, (2), 865-881.
38. Kong, L.; Feng, M.; Liu, Y.; Zhang, Y.; Zhang, C.; Li, C.; Qu, Y.; An, J.; Liu, X.; Tan, Q., Elucidating the pollution characteristics of nitrate, sulfate and ammonium in PM 2.5 in Chengdu, southwest China, based on 3-year measurements. *Atmos Chem Phys* **2020,** *20*, (19), 11181-11199.
39. Tao, J.; Zhang, L.; Cao, J.; Zhong, L.; Chen, D.; Yang, Y.; Chen, D.; Chen, L.; Zhang, Z.; Wu, Y., Source apportionment of PM2.5 at urban and suburban areas of the Pearl River Delta region, south China-with emphasis on ship emissions. *Sci. Total Environ.* **2017,** *574*, 1559-1570.
40. Ma, X.; Wang, L.; Ma, S.; Wei, Z.; Zhang, C.; Zheng, A., Spatial and temporal distribution and source analysis of components in PM2.5, Handan. *Environ. Chem.* **2017,** *36*, 1932-1940 (in Chinese with English abstract).
41. Du, W.; Zhang, Y.; Chen, Y.; Xu, L.; Chen, J.; Deng, J.; Hong, Y.; Xiao, H., Chemical characterization and source apportionment of PM2.5 during spring and winter in the Yangtze River Delta, China. *Aerosol Air Qual. Res.* **2017,** *17*, (9), 2165-2180.
42. Jiang, N.; Guo, Y.; Wang, Q.; Kang, P.; Zhang, R.; Tang, X., Chemical composition characteristics of PM2.5 in three cities in Henan, central China. *Aerosol Air Qual. Res.* **2017,** *17*, (10), 2367-2380.
43. Zhang, Z.; Hu, G.; Yu, R.; Hu, Q.; Liu, X., Characteristics and sources apportionment of water-soluble ions in PM2.5 of Xiamen City, China. *China Environ. Sci.* **2016,** *36*, (7), 1947-1954 (in Chinese with English abstract).
